# AI‐Enabled Imaging for Pathogen Detection Under Stress Conditions: A Systematic Review

**DOI:** 10.1111/1541-4337.70468

**Published:** 2026-04-22

**Authors:** MeiLi Papa, Gillian Kuehnle, Yoo Jung (Erika) Oh, Jiyoon Yi

**Affiliations:** ^1^ Department of Biosystems and Agricultural Engineering Michigan State University East Lansing Michigan USA; ^2^ Department of Communication Michigan State University East Lansing Michigan USA

## Abstract

Advances in pathogen detection that incorporate artificial intelligence (AI) may capture microbial signals under challenging environmental conditions that traditional methods miss. This systematic review evaluates the application, performance, and methodological characteristics of AI‐enabled imaging for pathogen detection, including its impact on speed, accuracy, and modeling under stress conditions. Studies were systematically identified from five electronic databases using search terms related to AI, pathogen, detection, and imaging. Inclusion criteria, defined using the Population, Intervention, Comparators, Outcome, Study design (PICOS) framework, focused on microscopy‐based pathogen detection enhanced by AI. Data extraction followed the Preferred Reporting Items for Systematic Reviews and Meta‐Analyses (PRISMA) guidelines and captured biological sample preparation, imaging modalities, AI‐enabled data analyses, comparator methods, and performance metrics. Of 2736 citations retrieved, 120 were reviewed in full and 28 studies met the inclusion criteria. These represented more than 40 pathogens, most commonly *Salmonella* spp. and *Escherichia coli*. Only three studies explicitly evaluated signals from stress or inactivated states. Comparator methods (e.g., culture‐based or molecular assays) were infrequently reported, limiting benchmarking against established workflows. Reporting inconsistencies in laboratory protocols and computational pipeline further complicated reproducibility and precluded meta‐analysis. Overall, this review offers a comprehensive overview of current AI‐enabled imaging approaches from both biological and computational perspectives and highlights the need for standardized benchmarks and reporting practices to support reproducible, transferable pathogen detection.

**Abbreviations**
AI: artificial intelligence
AUC: area under the curve
CMOS: complementary metal‐oxide semiconductor
CNN: convolutional neural network
EMCCD: electron‐multiplying charged‐coupled device
FN: false negative
FP: false positive
*k*‐NN: *k*‐nearest neighbors
LSTM: long short‐term memory
MIL: multiple instance learning
ML: machine learning
PCA: principal component analysis
PICOS: Population, Intervention, Comparators, Outcome, Study design
PRISMA: Preferred Reporting Items for Systematic Reviews and Meta‐Analyses
qPCR: quantitative polymerase chain reaction
RF: random forest
RNN: recurrent neural network
ROC: receiver operating characteristic
ROI: region of interest
RQ: research questions
QUADAS: Quality Assessment of Diagnostic Accuracy Studies
SNV: standard normal variate
SVM: support vector machine
TN: true negative
TP: true positive

## 1 Introduction

Developing advanced pathogen detection technologies is crucial for ensuring food safety, protecting public health, and enhancing environmental monitoring. However, each of these domains is characterized by inherent biosystems stressors that induce microbiological variability and hinder reliable pathogen detection without additional biological sample preparation or isolation. In food systems, diverse food matrices and processing conditions can interfere with detection methods, especially when pathogens are present at low concentrations (Wang and Salazar 2016; Acuff and Ponder 2020). Foodborne pathogens are also exposed to stressors such as refrigeration, acidification, osmotic shifts, or sanitizer residues, which may alter physiological states and affect detectability (Foddai and Grant 2020). In clinical settings, detection is influenced by variable host responses, co‐infections, and the presence of non‐viable pathogens (Bliven and Maurelli 2016; Vicente‐Santos et al. 2023). Environmental monitoring presents additional complexity due to heterogeneous samples such as water, soil, and air, where fluctuating conditions can impact pathogen survival and signal clarity (Dupont et al. 2020).

Traditional detection methods can be limited by delayed testing time and reduced sensitivity or specificity in complex samples (Law et al. 2015; Hameed et al. 2018). A central challenge is identifying biomarkers that reliably distinguish target pathogens from non‐infectious background material despite biological noise and variability (Cangelosi and Meschke 2014; Emerson et al. 2017; Zeng et al. 2016). In addition, distinguishing physiological states, including viable, viable but stressed, and non‐viable cells, adds another layer of complexity (Paniel and Noguer 2019). Culture‐based methods remain the gold standard for many applications, particularly in regulatory and confirmatory testing, but often require extensive sample preparation and time; molecular assays can detect nucleic acids from non‐viable cells and may not reflect physiological state (Guo et al. 2021; Pinto et al. 2015). Together, these limitations motivate approaches that minimize biological sample preparation while leveraging richer signals to improve detection performance and interpretability.

Optical microscopy has shown promise for rapid pathogen detection across food, clinical, and environmental contexts. Brightfield and darkfield imaging with Gram staining, as well as fluorescence microscopy with biomarkers, can capture cellular morphology and localization features relevant to classification (Kim et al. 2023; Borowa et al. 2021; He et al. 2018). Furthermore, multimodal approaches such as hyperspectral microscopy enable observation with reduced reliance on extensive sample preparation, while providing spectrochemical signatures related to intracellular components and metabolic activity (Zhu et al. 2014; Gowen et al. 2015; Gao and Smith 2015). Such richness may be valuable in complex matrices where natural biological background noise is strong and physiological states may vary (Y. W. Seo et al. 2013; Park et al. 2023). The real‐time capability of microscopy‐based detection offers advantages in public health, where rapid detection is crucial during outbreaks and for diagnostics by expediting identification, allowing for quicker response times (Akbari et al. 2012; Akgönüllü and Denizli 2022).

Incorporating artificial intelligence (AI) with microscopy has enabled automated feature extraction and classification, supporting faster analysis in imaging‐based pathogen detection (Pinto‐Coelho 2023). In food safety, machine learning (ML) and deep learning classification models have been applied to accelerate image analysis and classify foodborne pathogens (Kang et al. 2021; Ma et al. 2023; Yi et al. 2023). The integration of medical imaging techniques with AI has also demonstrated the ability to extract valuable insights and patterns that could not be readily discernable to the human eye (Pinto‐Coelho 2023). However, as these approaches are still emerging, consistent reporting of experimental protocols, computational pipelines, and evaluation methods is essential to support reproducibility and fair comparisons with conventional detection workflows.

Within the food science domain, AI and computer vision have been applied at multiple spatial scales, from macroscopic inspection of food quality to microscopic analysis of bacteria (e.g., single cells, colonies). Because this review focuses on pathogen detection, we restrict our scope to imaging modalities that spatially resolve bacterial cells or colonies and to AI models trained on these microbial signals. This does not cover non‐microbial applications such as surface defect detection, ripeness assessment, or bulk compositional imaging, which involve different imaging characteristics and do not directly address microbial hazards.

Therefore, this systematic review synthesizes techniques and methodological characteristics used in AI‐enabled imaging for bacterial pathogen detection and evaluates their relevance to food safety and related public health contexts. Because applications specifically within food systems remain limited and the field is still emerging, we define the scope more broadly as microscale imaging of bacterial pathogens across food, environmental, agricultural, and closely related biomedical settings, emphasizing approaches that are technically transferable to food safety.

Unlike narrative reviews, which qualitatively summarize literature selected through subjective criteria, systematic reviews follow a predefined, transparent process to identify, screen, and synthesize all relevant studies. This captures the full range of available evidence, including heterogeneous methods, offering a comprehensive view of research within a clearly scoped domain. Accordingly, this review adheres to the Preferred Reporting Items for Systematic Reviews and Meta‐Analyses (PRISMA) guidelines, widely used standards that structure systematic reviews into distinct Methods, Results, and Discussion sections to ensure transparency and reproducibility (Page et al. 2021). This process also aligns with the *IFIS Good Review Practice: A Researcher Guide to Systematic Review Methodology in the Sciences of Food and Health* (Kalantar and Hollier 2023), which emphasizes the role of systematic reviews in synthesizing evidence for decision makers, policy makers, and practitioners. Although first established in clinical research, systematic review methods are now widely adopted across scientific disciplines, including food and health sciences, to enable rigorous and unbiased evaluation of emerging research areas. By synthesizing AI‐enabled imaging studies alongside reported comparisons with traditional pathogen detection approaches, this review examines a critical gap in the literature. These insights aim to inform future research, promote consistent reporting practices, and support development of scalable and reliable systems for rapid pathogen detection across food safety, public health, and environmental monitoring contexts.

## 2 Methods

### 2.1 Research Questions

This systematic review rigorously evaluated the application, efficacy, and technological advancements of AI in imaging‐based pathogen detection by addressing the following research questions (RQs):

**RQ1**. Which AI algorithms have been employed in imaging for pathogen classification?
**RQ2**. How does AI‐enabled imaging enhance the speed and accuracy of pathogen detection and classification compared to traditional methods?
**RQ3**. What are the latest advancements in integrating AI with multimodal imaging techniques for pathogen research?
**RQ4**. How does AI facilitate early detection of pathogens under stress conditions?

### 2.2 Eligibility Criteria

The inclusion and exclusion criteria were determined using the Population, Intervention, Comparators, Outcome, Study design (PICOS) framework (Schardt et al. 2007), a commonly used approach for structuring eligibility criteria in systematic reviews, as detailed in Table S1. We included peer‐reviewed papers or full‐text conference proceedings that focused on bacterial detection by applying AI/ML techniques. The focus on original datasets was intended to ensure that studies provided full methodological descriptions and laboratory context, avoiding potential biases introduced by benchmark‐only datasets lacking biological provenance. In this review, we use “imaging” to refer to optical microscopy and related modalities that capture spatially resolved images of individual bacterial cells or colonies (e.g., brightfield, fluorescence, phase‐contrast, or hyperspectral microscopy), rather than bulk measurements or only global shape descriptors summarizing each object in images (e.g., area, perimeter, eccentricity). The latter discard most pixel‐level spatial structure that deep learning models can exploit when operating directly on full images. In addition, studies available in full‐text and written in English from 2012 to 2024 were selected, with 2012 chosen as the starting point to mark the onset of deep learning's rapid evolution driven by GPU acceleration (Shao et al. 2022). Studies were excluded if they focused on non‐bacterial substrates; used electron microscopy or non‐imaging methods that did not provide spatially resolved images of bacteria; relied on downloaded datasets; or examined only traditional detection methods without AI/ML integration. In addition, dissertations, theses, reviews, books or book chapters, conference abstracts without full text, editorials, news articles, posters, proposals, retracted publications, studies published outside the 2012–2024 timeframe, or non‐English publications were also excluded. These sources are often considered gray literature; therefore, this systematic review focuses on peer‐reviewed, full‐text research articles and proceedings.

### 2.3 Information Sources and Search Strategy

A combination of key search terms was used across five electronic databases (PubMed, IEEE, Scopus, Web of Science Core Collection, and ACM Digital Library) to identify studies on AI applications in imaging‐based pathogen detection. The searches were completed in May 2024. To build the search strategy, we first identified key terms and their potential synonyms to ensure comprehensive retrieval of relevant literature. Boolean operators (AND, OR) and truncation symbols were used to combine and broaden keyword searches. In accordance with PRISMA and IFIS guidance, the search strategy was intentionally broad to maximize sensitivity, with specificity achieved through PICOS‐based screening of titles/abstracts and full texts. The primary keywords and their alternative terms are as below:

- **AI**–ML, deep learning
- **Pathogen**–microbe, microorganism, microbial, microbiological, bacteria, bacterial
- **Detection**–diagnostics, classification, prediction, physiological signals, stress phenotype, resilience phenotype
- **Imaging**–image analysis, microscopy, microscope, hyperspectral, multispectral, multimodal

### 2.4 Study Selection

All references were imported into the Zotero reference management software. These references were then imported into the Covidence systematic review software (Veritas Health Innovation). Duplicates were identified and removed in Covidence prior to screening. Reviewers were not blinded to author, institution, or journal information during screening. Screening was conducted primarily by two independent reviewers (M.P. and G.K.), with discrepancies resolved by discussion with a third reviewer (J.Y.). Most discrepancies involved borderline cases, such as whether the target organism and imaging modality met the predefined eligibility criteria, and all were resolved by consensus. Before the full screening of the studies, three reviewers (M.P., G.K., and J.Y.) met to discuss the procedure for title and abstract screening using 10 randomly selected articles to ensure consistency between the reviewers.

### 2.5 Data Collection Process and Data Items

Data collection forms were developed based on the PRISMA guidelines (Page et al. 2021; Shamseer et al. 2015), so the data items were organized using the PICOS framework (Schardt et al. 2007). The data extraction form includes domains of (1) study characteristics (e.g., authors, year of publication, title, countries of study, study design), (2) pathogen characteristics (e.g., bacterial strains, bacterial sample types and origin, bacterial physiological states), (3) AI‐enabled detection method characteristics (e.g., biological sample preparation, biological replicates, imaging sample type, imaging modality, imaging parameters–intensity/exposure time/gain, data size for model training/test, data preprocessing methods, AI analysis methods, model architectures, test dataset source), (4) comparator characteristics (e.g., conventional detection methods, additional sample preparation, target detection level–colony/single‐cell/subcellular, detection limit), and (5) pathogen detection performance (e.g., evaluation metrics, detection limit, quantification capacity, overall testing time). Comparator information (e.g., culture‐based plating or other gold standard assays) was recorded when reported, as this is an essential PICOS component in systematic reviews and allows the performance of AI‐enabled imaging to be interpreted relative to existing practice under matched experimental conditions. Comparator methods were extracted as part of the data items and were not used as an inclusion criterion. Two reviewers (M.P. and G.K.) independently validated the extracted data. All data were extracted in duplicate by the two primarily reviewers, with discrepancies resolved by discussion with a third reviewer. The data extraction form was piloted on three randomly selected studies and refined for clarity and completeness before full‐scale extraction.

### 2.6 Methodological Risk of Bias Assessment

Selected studies were assessed by two independent reviewers (M.P. and G.K.) using a modified version of the Quality Assessment of Diagnostic Accuracy Studies 2 (QUADAS‐2) tool (Whiting et al. 2011). QUADAS‐2 is a standardized framework widely used in systematic reviews of diagnostic tests to evaluate risk of bias across four domains: patient recruitment, index test (the diagnostic test under evaluation), reference standard (the comparator or gold standard method), and flow and timing (per QUADAS‐2 terminology). For this systematic review, we adapted these domains to bacterial pathogen imaging by redefining them as: (1) pathogen selection, (2) index test (proposed AI‐enabled detection methods), (3) reference standard (conventional detection methods), and (4) flow and timing. The evaluation was based on 12 signaling questions (3 per domain) assessing potential methodological bias and reporting completeness, as detailed in Table S2. These signaling questions were adapted from the original QUADAS‐2 guidance by rephrasing biomedical concepts into microbial food safety contexts. Questions unrelated to our research context, such as those addressing responder rates or human participant selection, were excluded. Each signaling question was rated as “yes,” “no,” or “unclear,” with “unclear” assigned when insufficient information was reported to make a judgment. The scoring thresholds for high, unclear, and low risk of bias were pre‐specified before the assessment process began. Each study was classified into one of three categories, that is, “high risk of bias” (< 4 “yes” responses), “unclear” (4–8 “yes”), or “low risk of bias” (> 8 “yes”), following a commonly used approach in systematic reviews adapting the QUADAS‐2 tool (Whiting et al. 2011). The same criteria were applied to all studies, including those authored by our group. This assessment addresses methodological risk of bias within individual studies and is distinct from publication bias across all studies included in the review (see Section 2.7). Any disagreements between the two primary reviewers (M.P. and G.K.) were resolved through discussion with a third reviewer (J.Y.).

### 2.7 Synthesis of Results

Due to variability in study methods and sample selection, the results were synthesized qualitatively rather than via meta‐analysis. Since the included studies were highly heterogeneous in design, outcomes, and performance metrics, a formal statistical assessment of publication bias was not considered appropriate. Here, “publication bias” refers to potential bias across all the included studies, such as selective non‐publication or under‐reporting of studies with certain types of results (e.g., preferential publication of positive findings), which is typically assessed using methods like funnel plots or regression‐based tests. For transparency in the qualitative synthesis, full study citations were retained as primary identifiers in all summary tables, consistent with the systematic review conventions to ensure that all summarized data can be directly traced to their original sources.

## 3 Results

**FIGURE 1:**
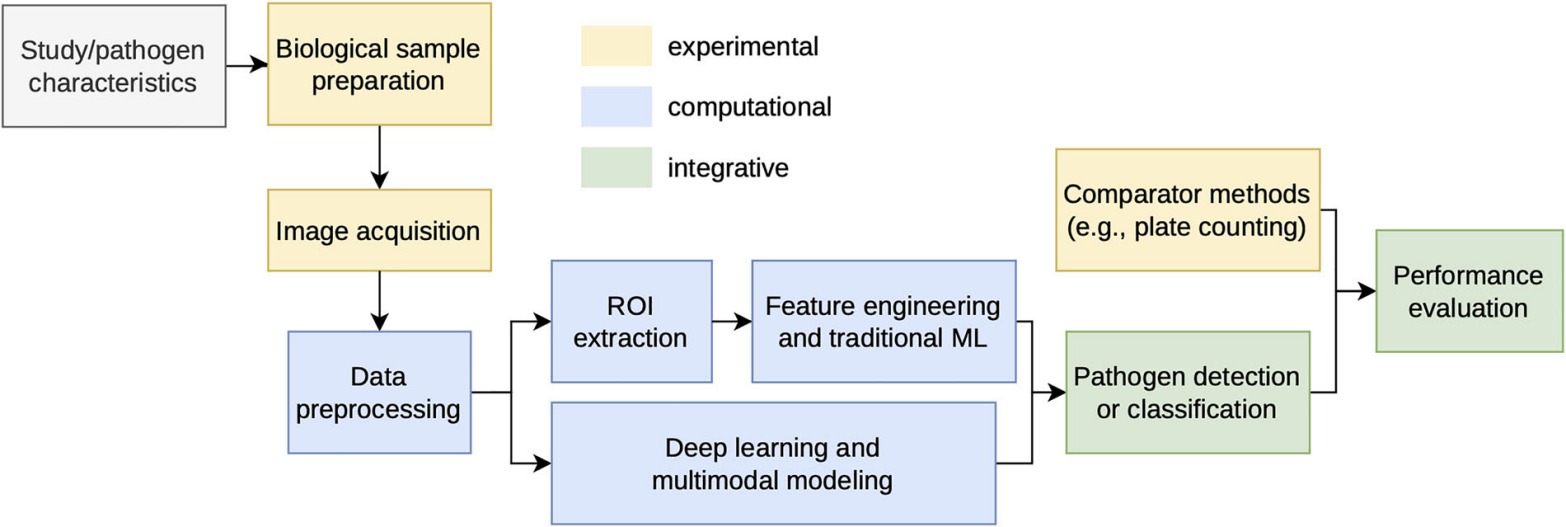
A schematic overview of the AI‐enabled imaging workflow synthesized from the 28 included studies, illustrating the progression from study and pathogen characteristics through biological sample preparation, image acquisition, computational modeling, pathogen detection or classification, comparator methods, and performance evaluation. Colors indicate experimental, computational, and integrative components.

Figure 1 provides a schematic overview of the AI‐enabled imaging workflow synthesized from the included studies. To orient readers from different disciplinary backgrounds, the Results section is organized around key workflow components: Table 1 summarizes study and pathogen characteristics (Section 3.1); Tables 2, 3, 4 summarize biological sample preparation, imaging modalities/parameters, and preprocessing/modeling approaches (Sections 3.2.2.1–3.2.2.3); Table S3 summarizes comparator methods (Section 3.2.3); and Table 5 summarizes evaluation metrics and detection performance (Section 3.2.4).

**TABLE 1:** Summary of study and pathogen characteristics.

No.	First author (year), country	Bacterial strains	Bacterial sample types and origin	Physiological states
1	Akbar (2021), Malaysia, Indonesia	*Burkholderia pseudomallei, Hemophilus influenzae, Klebsiella pneumoniae, Pseudomonas aeruginosa, Staphylococcus aureus, Streptococcus pneumoniae*	Laboratory strains (Parasitology Lab at University Malaysia Pahang)	Viable and culturable
2	Ali (2020), Germany	*E. coli* AG100 (derived from *E. coli* K12), *E. coli* strains (407, 416, 422, 455, 500, 544, 545, 554, 579, 673, 683)	Laboratory strain; clinical isolates (sepsis patients from Jena University Hospital)	Viable and culturable; antibiotic‐treated
3	Borowa (2021), Poland	*Klebsiella pneumoniae*	Clinical isolates (respiratory tract, urine, wound swabs, blood, fecal samples, catheter JJ, and urethral swab)	Viable and culturable
4	Borowa et al. (2023), Poland	*E. coli, Lactobacillus plantarum, Neisseria gonorrhoeae, Staphylococcus aureus*	Laboratory strains (ATCC)	Viable and culturable
5	Fong (2020), USA	*Campylobacter jejuni, E. coli, Listeria innocua, Listeria monocytogenes, Salmonella* Typhimurium, *Staphylococcus aureus*	Food processing isolates	Viable and culturable
6	Gu (2020), China	*E. coli, Salmonella* spp.*, Staphylococcus aureus*	Laboratory strains (Biological Lab of Animal Science School at Huazhong Agricultural University)	Viable and culturable
7	He (2018), China	*E. coli* MG1655, *Mycobacterium smegmatis* MC155, *Pseudomonas aeruginosa* PAO1	Laboratory strains	Viable and culturable
8	Hoorali (2020), Iran	*Bacillus anthracis*	Clinical samples (cutaneous anthrax disease patient tissues)	Non‐viable
9	Ibrahim (2021), Turkey	*Mycobacterium tuberculosis*	Clinical samples (sputum smears from tuberculosis patients, Istanbul Tuberculosis Control Association)	Non‐viable
10	Kang et al. (2020a), USA, China	*E. coli* serovars (O26, O45, O103, O111, O121, O145)	Laboratory strains (USDA FSIS culture collection)	Viable and culturable
11	Kang et al. (2020b), USA, China	*Campylobacter fetus, E. coli, Listeria innocua, Salmonella* Typhimurium, *Staphylococcus aureus*	Laboratory strain (ATCC); food isolates (chicken carcasses, USDA ARS PMSPRU)	Viable and culturable
12	Kang et al. (2020c), USA, China	*Campylobacter jejuni, E. coli, Listeria innocua, Salmonella* Typhimurium, *Staphylococcus aureus*	Laboratory strain (ATCC); food isolates (chicken rinsate, USDA ARS PMSPRU)	Viable and culturable
13	Kang (2021), USA, China	*Campylobacter jejuni, E. coli, Listeria innocua, Salmonella* Typhimurium, *Staphylococcus aureus*	Laboratory strain (ATCC); food isolate (chicken carcass rinses, USDA ARS PMSPRU)	Viable and culturable
14	Kim (2023), South Korea	*Cutibacterium acnes, Staphylococcus aureus*, *Staphylococcus epidermidis*	Laboratory strains (ATCC); NR	Viable and culturable
15	Ma (2023), USA	*E. coli* strains (LJH 1612, TVS 355, TVS 354, K‐12, ATCC 35218, ATCC 11775)	Laboratory strains (ATCC); food, environmental, animal, and clinical isolates (fresh produce, irrigation water, soil); *E. coli*‐spiked food sample (romaine lettuce)	Viable and culturable
16	Maeda (2018), Japan	*Pseudomonas aeruginosa, Staphylococcus aureus, Staphylococcus epidermidis, Staphylococcus haemolyticus, Staphylococcus saprophyticus, Staphylococcus simulans*	Laboratory strains (ATCC, NBRC)	Viable and culturable
17	Park (2023), USA	*E. coli, Listeria innocua, Salmonella* Enteritidis, *Salmonella* Heidelberg, *Salmonella* Typhimurium, *Staphylococcus aureus*	Laboratory isolates (USDA ARS PMSPRU)	Viable and culturable; dead
18	Rattray (2023), USA	*Pseudomonas aeruginosa*	Clinical and environmental isolates	Viable and culturable
19	Y. W. Seo (2013), USA	*Acinetobacter baumannii, Aeromonas salmonicida, Citrobacter koseri, Enterobacter cloacae, E. coli, Klebsiella oxytoca, Pseudomonas putida, Salmonella* Enteritidis, *Salmonella* Typhimurium, *Staphylococcus aureus*	Laboratory strains (USDA ARS PMSPRU); *Salmonella*‐spiked food samples (chicken carcass rinses)	Viable and culturable
20	Signoroni (2018), Italy	*E. coli*, *Enterococcus faecalis*, *Klebsiella pneumoniae*, *Proteus mirabilis*, *Proteus vulgaris*, *Pseudomonas aeruginosa*, *Staphylococcus aureus*, *Streptococcus agalactiae*	Laboratory strains (ATCC)	Viable and culturable
21	Spahn (2022), Germany, Portugal, UK, Finland	*Bacillus subtilis, E. coli, Staphylococcus aureus*	Laboratory strains	Viable and culturable
22	Tao (2023), China	*Acinetobacter baumannii, Burkholderia cepacian, Citrobacter afermentans, E. coli, Enterococcus faecalis, Enterococcus faecium, Klebsiella aerogenes, Klebsiella pneumoniae, Moraxella catarrhalis, Morganella morganii, Proteus mirabilis, Proteus vulgaris, Pseudomonas aeruginosa, Serratia marcescens, Staphylococcus aureus, Staphylococcus haemolyticus, Staphylococcus saprophyticus, Staphylococcus warneri, Stenotrophomonas maltophilia, Streptococcus anginosus, Streptococcus constellatus, Streptococcus salivarius* (62 strains)	Laboratory strains (ATCC); clinical isolates (urine, wound secretions, sputum, tissue fluid, articular cavity fluid, ascites, cerebrospinal fluid, purulence, lavage fluid, punctured fluid, drainage fluid, catheter, blood from the Second Affiliated Hospital of Air Force Military Medical University)	Viable and culturable
23	Treebupachatsakul (2019), Thailand	*Lactobacillus delbrueckii, Staphylococcus aureus*	Laboratory strains	Viable and culturable
24	Treebupachatsakul (2020), Thailand	*Lactobacillus delbrueckii, Micrococcus* spp*., Staphylococcus aureus*	Laboratory strains	Viable and culturable
25	Turra (2017), Italy	*E. coli*, *Enterococcus faecalis*, *Klebsiella pneumoniae*, *Proteus mirabilis*, *Proteus vulgaris*, *Pseudomonas aeruginosa*, *Staphylococcus aureus*, *Streptococcus agalactiae*	Laboratory strains (ATCC)	Viable and culturable
26	Wu (2024), China	*E. coli* (EPEC O26: K60, EPEC, CICC 10372)	Laboratory (China General Microbiological Culture Collection Center)	Viable and culturable; heat‐treated
27	Yi (2023), USA	*Bacillus subtillis, E. coli* BL21, *Listeria innocua, Pseudomonas fluorescens*	Laboratory strains (ATCC); *E. coli*‐spiked food and environmental samples (coconut water, spinach wash water, irrigation water)	Viable and culturable
28	H. Zhu (2023), China	*Bacillus subtilis, E. coli, Pseudomonas aeruginosa, Salmonella* spp., *Staphylococcus aureus*	Clinical isolates (urine, sputum, blood, tissue fluid)	Viable and culturable

Abbreviations: ATCC: American Type Culture Collection. NBRC: National Institute of Biotechnological Resource Center, Japan. PMSPRU: Poultry Microbiological Safety and Processing Research Unit. USDA ARS: US Department of Agriculture Agricultural Research Service. USDA FSIS: US Department of Agriculture Food Safety and Inspection Service.

**TABLE 2:** Summary of biological characteristics of AI‐enabled detection methods.

No.	Reference	Incubation (temperature/time)	Bacterial concentration	Imaging sample type	Other chemical process	Biological replicates
1	Akbar et al. (2021)	NR	NR	NR	Gram stain	NR
2	Ali et al. (2020)	37°C/90 min	5 × 10^5^ CFU/mL	Bacterial suspension (1 or 5 µL)	N/A	1–4
3	Borowa et al. (2021)	NR	NR	NR	Gram stain	2
4	Borowa et al. (2023)	37°C/overnight	NR	Colony suspension	Gram stain	2
5	Fong et al. (2020)	NR	NR	NR	N/A	NR
6	Gu et al. (2020)	37°C/20 ± 2 h	10^2^–10^4^ CFU/mL	Colony	N/A	NR
7	He et al. (2018)	NR/overnight	NR	Bacterial suspension (3 µL)	Fluorescence staining	NR
8	Hoorali et al. (2020)	N/A	N/A	Tissue	Methanol fixation, Giemsa staining	200
9	Ibrahim et al. (2021)	N/A	N/A	Sputum	Acid‐fast staining	100
10	Kang et al. (2020a)	NR	NR	Bacterial suspension (3 µL)	N/A	NR
11	Kang et al. (2020b)	37°C/16–48 h	NR	Bacterial suspension (3 µL)	N/A	10
12	Kang et al. (2020c)	37°C/24–48 h	NR	Bacterial suspension (3 µL)	N/A	NR
13	Kang et al. (2021)	37°C/24–48 h	NR	Bacterial suspension (3 µL)	N/A	NR
14	Kim et al. (2023)	37°C/overnight	NR	Bacterial suspension	Fluorescence staining	NR
15	Ma et al. (2023)	37°C/3 h	10^4^–10^7^ CFU/mL	Microcolony	N/A	9
16	Maeda et al. (2018)	37°C/overnight	3.2 × 10^5^ cells/mL	Bacterial suspension (1 µL)	N/A	NR
17	Park et al. (2023)	37°C/18–24 h 121°C/15 min (dead)	NR	Bacterial suspension (3 µL)	N/A	3
18	Rattray et al. (2023)	37°C/72 h	NR	Colony	N/A	4
19	Y. W. Seo et al. (2013)	35°C/18–24 h	10–10^8^ CFU/mL	Colony	N/A	5
20	Signoroni et al. (2018)	NR/18 h	NR	Colony	N/A	NR
21	Spahn et al. (2022)	37°C/overnight	NR	Bacterial suspension	Fluorescence staining	NR
22	Tao et al. (2023)	35°C–37°C/24–48 h	NR	Bacterial suspension	N/A	NR
23	Treebupachatsakul and Poomrittigul (2019)	30°C or 37°C/24 h	NR	NR	Gram staining	NR
24	Treebupachatsakul and Poomrittigul (2020)	30°C/24 h, 37°C/48 h	NR	NR	Gram staining	NR
25	Turra et al. (2017)	NR/18 h	NR	Colony	N/A	NR
26	Wu et al. (2024)	37°C/14–16 h	NR	Bacterial suspension (2.5 µL)	N/A	NR
27	Yi et al. (2023)	37°C/4 h	10–10^3^ CFU/mL	Bacterial suspension (100 µL), liquid	Phage infection, fluorescence staining	5
28	H. Zhu et al. (2023)	NR/48 h	10^8^/mL	Bacterial suspension (10 µL)	N/A	NR

Abbreviations: CFU: Colony forming units. N/A: Not applicable. NR: Not reported.

**TABLE 3:** Summary of imaging characteristics of AI‐enabled detection methods.

No.	Reference	Modality	Equipment	Illumination	Light source	Objective	Gain/ exposure
1	Akbar et al. (2021)	Optical microscopy	Olympus BX40 microscope	Brightfield	NR	NR	NR
2	Ali et al. (2020)	Optical microscopy	Axiobserver.Z1 microscope, scientific CMOS camera	Brightfield	NR	63×	NR
3	Borowa et al. (2021)	Optical microscopy	Olympus XC31 upright microscope, CMOS camera	Brightfield	NR	NR	NR
4	Borowa et al. (2023)	Optical microscopy	Olympus BX63 microscope, CCD camera	Brightfield	NR	100× oil	NR
5	Fong et al. (2020)	Hyperspectral microscopy	Olympus BX43 microscope, HinaLea 4200M camera	Darkfield	Tungsten halogen	NR	NR
6	Gu et al. (2020)	Hyperspectral imaging (non‐microscopic)	Specim V10E‐CL camera	Reflected	Halogen	N/A	NR/100 ms
7	He et al. (2018)	SIM	Nikon N‐SIM microscope, EMCCD camera	Fluorescence (laser‐based, SIM)	Laser (488 nm)	100× oil, TIRF	100×/50–100 ms
8	Hoorali et al. (2020)	Optical microscopy	Olympus microscope, camera	Brightfield	NR	NR	NR
9	Ibrahim et al. (2021)	Optical microscopy	NR	Brightfield	NR	NR	NR
10	Kang et al. (2020a)	Hyperspectral microscopy	Nikon Eclipse e80i upright microscope, EMCCD camera, AOTF spectrometer	Darkfield	Metal halide	100× oil	3.5%/250 ms
11	Kang et al. (2020b)	Hyperspectral microscopy	Nikon Eclipse e80i microscope, EMCCD camera, AOTF spectrometer	Darkfield	Metal halide	100× oil	1.6%/250 ms
12	Kang et al. (2020c)	Hyperspectral microscopy	Nikon Eclipse 80i microscope, EMCCD camera, AOTF spectrometer	Darkfield	Metal halide	100× oil	3.5%/250 ms
13	Kang et al. (2021)	Hyperspectral microscopy	Nikon Eclipse 80i microscope, EMCCD camera, AOTF spectrometer	Darkfield	Metal halide	100× oil	3.5%/250 ms
14	Kim et al. (2023)	Super‐resolution fluorescence microscopy	Custom‐built Nikon Ti2‐U inverted microscope	Fluorescence	NR	NR	NR
15	Ma et al. (2023)	Phase contrast microscopy	Olympus X71 inverted microscope, CCD camera	Phase contrast	Halogen	60× Ph	NR
16	Maeda et al. (2018)	Lensless imaging	CMOS sensor	Transmitted	Blue LED	N/A	NR/56 ms
17	Park et al. (2023)	Hyperspectral microscopy	Nikon Eclipse 80i microscope, EMCCD camera, AOTF spectrometer	Darkfield	Metal halide	100× oil	15 dB/250 ms
18	Rattray et al. (2023)	Optical microscopy	Nikon Eclipse Ti inverted microscope, CCD camera	Brightfield	NR	4×	NR
19	Y. W. Seo et al. (2013)	Hyperspectral imaging (non‐microscopic)	Custom VNIR hyperspectral imaging system	NR	NR	N/A	NR
20	Signoroni et al. (2018)	Hyperspectral imaging (non‐microscopic)	Specim V10E camera	Reflected	Halogen	N/A	NR
21	Spahn et al. (2022)	Optical or fluorescence microscopy, SIM	Nikon Eclipse Ti inverted microscope, Deltavision OMX system, Leica SP8, Nikon Ti‐E, EMCCD or scientific CMOS camera	Brightfield, fluorescence (laser‐based)	Halogen, lasers (488, 561 nm)	60×–100× oil, TIRF	NR
22	Tao et al. (2023)	Hyperspectral microscopy	Olympus BX43 microscope, Basler hyperspectral sensor	Transmitted	Halogen	100× oil	NR
23	Treebupachatsakul and Poomrittigul (2019)	Optical microscopy	Optika B‐292 microscope, CMOS sensor	Brightfield	NR	100× oil	NR
24	Treebupachatsakul and Poomrittigul (2020)	Optical microscopy	Optika B‐292 microscope or Olympus CX31 upright microscope, CMOS sensor	Brightfield	NR	100× oil	NR
25	Turra et al. (2017)	Hyperspectral imaging (non‐microscopic)	Specim V10E camera	Reflected	Halogen	N/A	NR
26	Wu et al. (2024)	Hyperspectral microscopy	Nikon Eclipse 100i upright microscope, EMCCD camera, SOC‐70 spectrometer	NR	Metal halide	100× oil	NR/99 ms
27	Yi et al. (2023)	Fluorescence microscopy	Olympus IX‐71 inverted microscope, CCD camera	Fluorescence	NR	100×	NR
28	H. Zhu et al. (2023)	Hyperspectral microscopy	Custom hyperspectral microscope imaging system	Transmitted	White LED	40×	NR

AOTF: Acousto‐optic tunable filter. CCD: Charge‐coupled device. CMOS: Complementary metal‐oxide‐semiconductor. EMCCD: electron‐multiplying CCD. LED: Light‐emitting diode. N/A: Not applicable. NR: Not reported. SIM: Structured illumination microscope. TRIF: total internal reflection fluorescence. VNIR: Visible and near‐infrared.

**TABLE 4:** Summary of data and AI analysis characteristics of AI‐enabled detection methods.

No.	Reference	Data type	Data size (train/test)	Preprocessing	AI analysis method	Models
1	Akbar et al. (2021)	Image	31,490/13,495	Resizing, augmentation	Classification	Proposed: CNN (DensNet‐201); Others: CNN (ResNet‐18, VGG‐19)
2	Ali et al. (2020)	Patch	NR	Resizing, CLAHE, ROI segmentation (U‐Net), patching	Segmentation, classification	CNN (U‐Net) PCA‐ML (OCSVM)
3	Borowa et al. (2021)	Patch	NR	Resizing, patching, filtering, normalization, augmentation	Classification	CNN (ResNet‐18) with MIL framework
4	Borowa et al. (2023)	Patch	47,149 total	Resizing, patching, filtering, normalization, augmentation	Classification	CNN (ResNet‐18) with MIL framework
5	Fong et al. (2020)	ROI	NR/100	ROI segmentation (*k*‐means, SAM), grayscale conversion, normalization, thresholding, shape descriptor extraction, SNV	Classification	Proposed: Multimodal network with RNN (LSTM) and CNN (1D‐CNN, ResNet); Others: PCA‐ML (SVM, RF, *k*‐NN)
6	Gu et al. (2020)	ROI	916/2210	Resizing, ROI segmentation (thresholding), filtering, multiplicative scatter correction, wavelength selection	Classification	Statistical (PLS‐DA), ML (SVM)
7	He et al. (2018)	ROI	2932 total	ROI segmentation (watershed), resizing	Classification	PCA‐ML (SVM, RF, *k‐*NN)
8	Hoorali et al. (2020)	Patch	1021/281	Patching, standardization, augmentation	Segmentation	CNN (UNet, UNet ++)
9	Ibrahim et al. (2021)	Image	124/54, 371/157, 367/159	Resizing, augmentation	Classification	CNN (AlexNet)
10	Kang et al. (2020a)	ROI	Ranging from 500/100 to 50,000/10,000	ROI segmentation (thresholding), SNV, wavelength selection	Classification	PCA‐statistical (LDA, SR), PCA‐ML (SVM), ANN (SAE)‐statistical (LDA, SR), ANN (SAE)‐ML (SVM)
11	Kang et al. (2020b)	ROI	200/100	Resizing, augmentation, ROI segmentation (U‐Net), SNV	Segmentation, classification	Proposed: CNN (U‐Net and 1D‐CNN); Others: PCA‐ML (SVM, *k*‐NN)
12	Kang et al. (2020c)	ROI	4500/500	ROI segmentation (manual single‐cell selection), normalization, shape descriptor extraction, SNV	Classification	Proposed: Multimodal network with RNN (LSTM) and CNN (ResNet, 1D‐CNN); Others: PCA‐ML (SVM, RF, *k*‐NN)
13	Kang et al. (2021)	ROI	900/100	ROI segmentation (thresholding), SNV, normalization, outlier detection	Classification	Proposed: RNN (LSTM); Others: PCA‐ML (LDA, SVM, *k*‐NN)
14	Kim et al. (2023)	Image	NR/253, NR/114	NR	Classification	CNN (ResNet‐18, RegNetY‐16GF, EfficientNet‐V2‐S, SwinV2‐S, SwinV2‐T)
15	Ma et al. (2023)	Image	1764/756	Resizing, bounding box annotation, augmentation	Object detection	CNN (YOLOv4)
16	Maeda et al. (2018)	ROI	NR/125	Contrast enhancement, color inversion, ROI segmentation (Otsu thresholding), discriminative parameter extraction	Classification	Statistical (LDA), ML (SVM, RF, *k*‐NN, naïve Bayes), ANN
17	Park et al. (2023)	ROI	1083/191	ROI segmentation (U‐Net), shape descriptor extraction, SNV	Classification	Multimodal CNN (1D‐CNN and ResNet)
18	Rattray et al. (2023)	Image	266/69	Resizing, normalization, shape descriptor extraction, augmentation	Classification	Proposed: CNN (ResNet‐50, VGG‐19, MobileNetV2, Xception) Other: ML (SVM)
19	Y. W. Seo et al. (2013)	ROI	NR	Savitzky–Golay smoothing, ROI segmentation (manual colony selection)	Classification	Statistical (LDA, QDA, Mahalanobis distance), PCA‐statistical, ML (SVM, *k*‐NN), PCA‐ML
20	Signoroni et al. (2018)	ROI	11,649/4993	Flat‐field correction, Savitzky–Golay smoothing, ROI segmentation (watershed)	Classification	Proposed: 1D‐CNN; Others: PCA‐ML (SVM, RF)
21	Spahn et al. (2022)	Patch or ROI	Small‐to‐moderate (e.g., 28/5, 94/5)	Resizing, patching, contrast enhancement, augmentation, ROI segmentation (manual selection, thresholding, U‐Net)	Segmentation, object detection, denoising, digital labeling, super‐resolution image prediction	CNN (U‐Net, U‐Net variants, YOLOv2), GAN (Pix2pix)
22	Tao et al. (2023)	ROI	119,000/51,000, 630/1470	Manual single‐cell cropping, flat‐field correction, ROI segmentation (*k*‐means), normalization	Classification	Proposed: Multimodal CNN; Others: CNN (1D‐CNN, ResNet, DenseNet, multimodal network)
23	Treebupachatsakul and Poomrittigul (2019)	Image	320/80	NR	Classification	CNN (LeNet)
24	Treebupachatsakul and Poomrittigul (2020)	Image	160/40	NR	Classification	CNN (LeNet)
25	Turra et al. (2017)	ROI	11,649/4993	Flat‐field correction, Savitzky–Golay smoothing, ROI segmentation (watershed)	Classification	Proposed: 1D‐CNN; Others: PCA‐ML (SVM, RF)
26	Wu et al. (2024)	ROI	1050/450	Contrast enhancement, ROI segmentation (edge detection, morphological filtering), Savitzky–Golay smoothing, normalization, augmentation	Classification	PCA‐statistical (DA), PCA‐ML (SVM, RF, *k‐*NN), PCA‐RNN (LSTM)
27	Yi et al. (2023)	Image	342/114–164	Resizing, normalization, keypoint annotation, augmentation	Object detection	CNN (Faster R‐CNN)
28	H. Zhu et al. (2023)	ROI	NR	ROI segmentation (edge detection, morphological filtering), normalization	Classification	PCA‐ML (SVM)

Abbreviations: ANN: (Artificial) neural network. CLAHE: Contrast limited adaptive histogram equalization algorithm (contrast enhancement). CNN: Convolutional neural network. DA: Discriminant Analysis. GAN: Generative adversarial network. *k*‐NN: *k*‐nearest neighbors. LDA: Linear discriminant analysis. LSTM: long short‐term memory. MIL: multiple instance learning. ML: machine learning (traditional). NR: Not reported. OCSVM: Once‐class SVM. PCA: principal component analysis. PLS‐DA: Partial least squares discriminant analysis. QDA: Quadratic discriminant analysis. RF: random forest. RNN: recurrent neural network. ROI: region of interest. SAE: Stacked autoencoder. SAM: Spectral angle mapping. SNV: standard normal variate. SR: Softmax regression. SVM: support vector machine.

**TABLE 5:** Summary of pathogen detection performance of AI‐enabled detection methods.

No.	Reference	Accuracy (%)	Sensitivity (%)	Specificity (%)	AUC (%)	Other metrics
1	Akbar et al. (2021)	99.24	99.20	99.87	NR	FPR
2	Ali et al. (2020)	NR	41.07–91.6	NR	72–83	N/A
3	Borowa et al. (2021)	56.3–64.8	56.3–64.8	NR	68.3–78.6	Precision, F1 score
4	Borowa et al. (2023)	60.3–96.0	NR	NR	69.8–98.7	N/A
5	Fong et al. (2020)	91.0–98.4	NR	NR	NR	N/A
6	Gu et al. (2020)	NR	NR	NR	NR	Confusion matrix only
7	He et al. (2018)	96.83–98.36	NR	NR	NR	F1 score
8	Hoorali et al. (2020)	81–97	84–98	80–97	NR	Precision, DSC
9	Ibrahim et al. (2021)	98.09–98.73	96.77–99.20	97.67–100	NR	N/A
10	Kang et al. (2020a)	75.3–94.9	75–95	NR	NR	Precision, F1 score
11	Kang et al. (2020b)	90	NR	NR	NR	N/A
12	Kang et al. (2020c)	91.0–98.4	NR	NR	99–100	N/A
13	Kang et al. (2021)	90.4–92.9	NR	NR	NR	N/A
14	Kim et al. (2023)	79.8–97.9	NR	NR	NR	N/A
15	Ma et al. (2023)	NR	87.4–100	NR	NR	IoU, precision, AP, mAP
16	Maeda et al. (2018)	74.4–100.0	64.0–100.0	86.0–100.0	NR	PPV
17	Park et al. (2023)	64.13–100	NR	NR	NR	N/A
18	Rattray et al. (2023)	62.75–90.73	NR	NR	NR	N/A
19	Y. W. Seo et al. (2013)	80.5–100	NR	NR	NR	Kappa coefficient
20	Signoroni et al. (2018)	99.7	NR	NR	NR	N/A
21	Spahn et al. (2022)	NR	36–100	NR	NR	IoU, precision, mAP, SSIM, PSNR
22	Tao et al. (2023)	92	NR	NR	99.09–99.97	Kappa coefficient
23	Treebupachatsakul and Poomrittigul (2019)	NR	NR	NR	NR	Train accuracy only
24	Treebupachatsakul and Poomrittigul (2020)	NR	NR	NR	NR	Train accuracy only
25	Turra et al. (2017)	99.7	NR	NR	NR	N/A
26	Wu et al. (2024)	65.56–98.22	NR	NR	NR	N/A
27	Yi et al. (2023)	80–100	NR	NR	NR	N/A
28	H. Zhu et al. (2023)	93.6	93.6	98.4	97–100	Precision

Abbreviations: AP: Average precision. DSC: Dice similarity coefficient. FPR: False positive ratio. IoU: Intersection of union. mAP: mean average precision. N/A: Not applicable. NR: Not reported. PPV: Positive predictive value. PSNR: Peak‐signal‐to‐noise ratio. SSIM: structural similarity.

### 3.1 Study Selection

**FIGURE 2:**
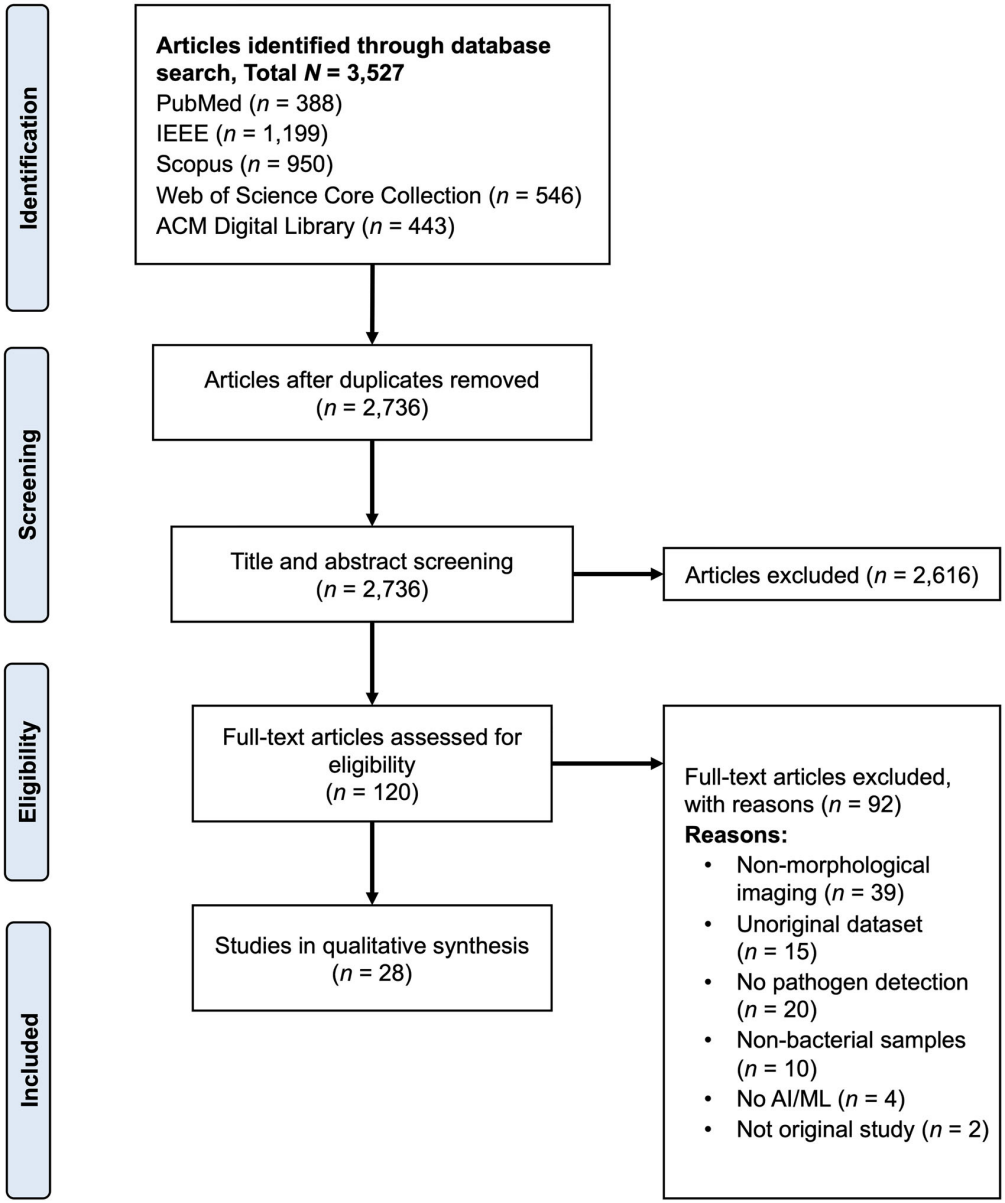
Flow diagram illustrating the article screening and selection process for this systematic review.

Figure 2 presents the PRISMA flow diagram summarizing study identification, screening, eligibility, and inclusion. The initial database search retrieved 3527 records from five electronic databases (PubMed, IEEE, Scopus, Web of Science Core Collection, and ACM Digital Library). After removing 791 duplicates, 2736 unique records proceeded to title and abstract screening using the predefined PICOS‐based inclusion and exclusion criteria (Table S1). Two reviewers (M.P. and G.K.) independently conducted the screening, achieving a high inter‐reviewer agreement rate of 98.9%, and excluded 2616 studies primarily for unrelated modalities, non‐bacterial targets, or lack of AI/ML integration. The remaining 120 full‐text articles underwent eligibility assessment (89.9% agreement), with discrepancies resolved by a third reviewer (J.Y.). Of these, 92 studies were excluded for reasons shown in Figure 2, most frequently because they did not include spatially resolved imaging of bacterial cells or colonies (*n* = 39). Other reasons included not focusing on pathogen detection (*n* = 20), using non‐original datasets (*n* = 15), use of non‐bacterial samples (*n* = 10), absence of AI/ML methodology (*n* = 4), or being non‐original studies (*n* = 2). Since the same PICOS‐based eligibility criteria were applied at both the title/abstract and full‐text stages, some exclusion reasons appear at both levels, with full‐text review providing a more detailed assessment than was possible from titles and abstracts alone. In total, 28 studies met all criteria and were included in the qualitative synthesis.

### 3.2 Main Findings

#### 3.2.1 Study and Pathogen Characteristics

The 28 included studies had been published between 2013 and 2024. Table 1 provides detailed characteristics of each study. Following the systematic review reporting conventions, the first column numbers the included studies, and the second column lists the study citation. This identifier format is used consistently in all results summary tables to maintain study‐level traceability. Of these, 10 were conducted in the United States and the remaining 18 were conducted in China, Italy, Poland, Thailand, Korea, Malaysia, Indonesia, Iran, United Kingdom, Portugal, Finland, Germany, and Japan. This distribution underscores the global interest in AI‐enabled pathogen detection, although representation from low‐ and middle‐income countries remains limited, which may influence the diversity of strains studied and the generalizability of findings to underrepresented regions.

Most studies (*n* = 26) focused on viable and culturable bacteria grown under optimal laboratory conditions, reflecting the experimental control and reproducibility advantages of such systems. Only three studies explicitly examined bacteria under stress or inactivated conditions, including predictive modeling of antibiotic susceptibility in *Escherichia coli* (Ali et al. 2020), differentiation of live bacteria from dead ones (Park et al. 2023), and identification of active and inactivated states of *E. coli* following different levels of heat treatment (Wu et al. 2024). In terms of strain origin, 17 studies relied exclusively on readily available laboratory strains, such as ATCC or culture collection isolates, whereas the other 11 incorporated real‐world samples from clinical and environmental contexts. Among these, eight studies analyzed clinical samples (e.g., patient sputum, wound swabs, urine), while three studies included environmental or food‐associated samples (e.g., irrigation water, chicken carcass rinses, romaine lettuce) (Ali et al. 2020; Borowa et al. 2021; Hoorali et al. 2020; Ibrahim et al. 2021; Kang et al. 2021; Rattray et al. 2023; Kim et al. 2023; Ma et al. 2023; Tao et al. 2023; Yi et al. 2023; H. Zhu et al. 2023). Notably, a few studies integrated both laboratory and real‐world strains, offering a broader performance assessment across strain variability. However, the relatively small proportion of environmental or clinical sample‐based work limits insight into AI performance under heterogeneous and potentially noisy conditions.

Collectively, the included studies covered 40 distinct microbial species, dominated by Gram‐negative bacteria (*n* = 22 species), including key foodborne pathogens such as *E. coli* (multiple strains and serovars), *Salmonella* spp., and *Pseudomonas aeruginosa*. Gram‐positive bacteria (*n* = 14 species) were also represented, with *Staphylococcus aureus*, *Listeria innocua*, and *Bacillus subtilis* among the most common. Other notable inclusions were opportunistic or high‐consequence pathogens such as *Burkholderia pseudomallei* and *Mycobacterium tuberculosis*, although these were comparatively rare in the dataset. The dominance of *E. coli* across both laboratory and applied studies reflects its role as both a model organism and a major target for detection in food safety and clinical microbiology.

Overall, Table 1 reveals that AI‐enabled imaging research to date remains heavily weighted toward controlled, laboratory‐based evaluations of common bacterial pathogens. Limited representation of stressed, rare, or clinically challenging organisms points to an important opportunity for expanding experimental scope to better capture the complexity of real‐world detection environments.

#### 3.2.2 AI‐Enabled Detection Method Characteristics

##### 3.2.2.1 Biological Sample Preparation

Table 2 summarizes biological sample preparation methods used across the 28 included studies, highlighting considerable heterogeneity in incubation time, temperature, bacterial concentration, and sample type prior to image data collection. Most studies used overnight or multi‐day incubation (up to 72 h) at 37°C to promote optimal growth for mesophilic pathogens or simulate human host conditions. Others extended incubation to 24–72 h to accommodate slower‐growing species or specialized treatments, with precise timings, such as 90 min, 3 h, 4 h, 20 h, 24 h, 48 h, or 72 h, reported for targeted experimental conditions (Ali et al. 2020; Gu et al. 2020; Ma et al. 2023; Rattray et al. 2023; Signoroni et al. 2018; Treebupachatsakul and Poomrittigul 2019, 2020; Yi et al. 2023; H. Zhu et al. 2023). Reported time ranges (e.g., 24–48 h) suggest flexibility of protocols or adaptation to specific bacterial growth rates, but also complicate direct comparisons across studies (Kang et al. 2020a, 2020b, 2021; Park et al. 2023; Y. W. Seo et al. 2013; Tao et al. 2023; Wu et al. 2024). A smaller subset used alternative incubation temperatures, 30°C and 35°C, to suit environmental isolates or strain‐specific requirements (Y. W. Seo et al. 2013; Treebupachatsakul and Poomrittigul 2019, 2020). Some studies reported temperature ranges, such as 35°C–37°C, to allow flexibility in experimental setups (Tao et al. 2023), while the others did not specify temperatures.

Bacterial concentrations used for image collection also showed significant variation. Among the included studies, only seven studies quantified bacterial concentration, with reported values spanning six orders of magnitude (10–10^8^ CFU/mL), typically in pure laboratory culture contexts (Ali et al. 2020; Gu et al. 2020; Ma et al. 2023; Maeda et al. 2018; Y. W. Seo et al. 2013; Yi et al. 2023; H. Zhu et al. 2023). The absence of such data in the remaining 21 studies indicates a persistent gap in standardizing and reporting inoculum density, a parameter critical for reproducibility and for understanding detection limits of AI‐enabled imaging.

Imaging sample type also varied widely, including bacterial suspension, liquid, microcolony, colony, tissue, or sputum. Bacterial suspensions (often 1–3 µL applied to slides) were most common, likely due to their simplicity and compatibility with high‐resolution imaging. Larger volumes (10–100 µL) appeared in studies aiming for broader field‐of‐view or lower magnification. Microcolony or colony samples also facilitated direct visualization of bacterial appearance. Less common sample types, such as tissue or sputum, were important for modeling clinically or environmentally relevant scenarios, but remain underrepresented, with only two studies each. Chemical pretreatments ranged from Gram and acid‐fast staining to fluorescence labeling and methanol fixation, with some protocols adding specialized steps (e.g., phage infection in Yi et al. 2023). However, several studies provided no details on chemical processing, raising concerns about transparency. Similarly, the number of biological replicates was frequently unreported (NR), further limiting reproducibility and meta‐analytic integration.

Overall, Table 2 shows that biological preparation protocols for AI‐enabled pathogen detection vary widely across studies, reflecting differences in target organisms, laboratory settings, and experimental objectives. While such variability is expected in an emerging, multidisciplinary field, incomplete or inconsistent reporting of core parameters such as bacterial concentration, replicate number, and slide preparation volume makes it difficult to replicate experiments and to evaluate how biological preparation influences AI model performance.

##### 3.2.2.2 Imaging Modality and Parameters

The 28 included studies utilized a diverse range of imaging modalities, reflecting differing experimental goals, bacterial targets, and analytical requirements (Table 3). Brightfield imaging was frequently paired with Olympus upright microscopes (BX40, BX63, XC31, or BX43 series) or Nikon Eclipse systems (e.g., Ti inverted, Ti‐E), with imaging sensors ranging from electron‐multiplying charge‐coupled device (EMCCD) and complementary metal‐oxide semiconductor (CMOS) cameras to scientific CMOS units. A small number used phase contrast (Ma et al. 2023, with an Olympus X71 inverted microscope and 60× phase objective) for enhanced contrast of cellular structures and colony boundaries.

Hyperspectral imaging was used in both microscopic and non‐microscopic formats, capturing spectral information at each pixel to analyze the biochemical composition and structural details of bacterial samples. This approach offers richer spectral and spatial data than conventional microscopy, which relies on a limited number of channels for spatial information. Hyperspectral microscopy was commonly performed with Nikon Eclipse 80i or 100i upright microscopes equipped with EMCCD cameras and acousto‐optic tunable filters (AOTFs), often illuminated by metal halide light sources (Kang et al. 2020a, 2020b, 2020c, 2021; Park et al. 2023; Wu et al. 2024). Objectives were typically 100× oil‐immersion to resolve single‐cell features, and exposure times of ∼250 ms with gain settings from 1.6% (Kang et al. 2020b) to 3.5% (Kang et al. 2020c, 2021) or 15 dB (Park et al. 2023) were reported. Tao et al. (2023) employed hyperspectral microscopy with an Olympus BX43 and a Basler hyperspectral sensor under transmitted halogen light, also at 100× oil. Non‐microscopic hyperspectral systems included the Specim V10E (Gu et al. 2020; Signoroni et al. 2018; Turra et al. 2017) and a custom VNIR system (Y. W. Seo et al. 2013), all operating in reflected light configurations with halogen illumination.

Fluorescence‐based approaches spanned super‐resolution methods (Kim et al. 2023) and structured illumination microscopy (SIM; He et al. 2018; Spahn et al. 2022). These used systems such as the Nikon N‐SIM with EMCCD, Deltavision OMX, Leica SP8, or custom‐built Nikon Ti2‐U platforms, with oil‐immersion objectives (60×–100×) and specialized illumination sources matched to the fluorophores, including lasers at 488, 561 nm, or total internal reflection fluorescence (TIRF) excitation. Yi et al. (2023) also used fluorescence microscopy with an Olympus IX‐71 inverted microscope at 100×.

Other specialized modalities included lens‐less imaging (Maeda et al. 2018), in which a CMOS sensor and blue LED provided wide‐field, transmitted light images at 56 ms exposure, and single‐molecule localization compatible systems like the SIM in He et al. (2018), which operated at 50–100 ms exposure for laser‐based fluorescence imaging.

Illumination sources varied by technique: halogen lamps were used in at least seven studies (Fong et al. 2020; Gu et al. 2020; Ma et al. 2023; Signoroni et al. 2018; Spahn et al. 2022; Tao et al. 2023; Turra et al. 2017), metal halide in six studies (Kang et al. 2020a, 2020b, 2020c, 2021; Park et al. 2023; Wu et al. 2024), white or blue LEDs in two studies (Maeda et al. 2018; H. Zhu et al. 2023), and lasers in two (He et al. 2018; Spahn et al. 2022). Some fluorescence‐based studies did not specify light source details (Kim et al. 2023; Yi et al. 2023), which can limit reproducibility.

Magnifications ranged from 4× (colony‐level brightfield imaging; Rattray et al. 2023) to 100× oil (Borowa et al. 2023; He et al. 2018; Kang et al. 2020a, 2020b, 2020c, 2021; Park et al. 2023; Spahn et al. 2022; Tao et al. 2023; Treebupachatsakul and Poomrittigul 2019, 2020; Wu et al. 2024), with intermediate values such as 40× (H. Zhu et al. 2023) and 60× phase (Ma et al. 2023). Imaging parameters, such as exposure time and gain, though not consistently reported, were detailed in nine studies (Gu et al. 2020; He et al. 2018; Kang et al. 2020a, 2020b, 2020c, 2021; Maeda et al. 2018; Park et al. 2023; Wu et al. 2024). Exposure times of 250 ms and shorter durations, such as 56 ms, were mentioned, which likely reflect efforts to balance image clarity with minimizing motion blur in capturing bacteria under low‐light conditions. Gain settings, recorded in Kang et al. (2020b) and Park et al. (2023) were as low as 1.6% or 16 dB, and as high as 3.5% in Kang et al. (2020c) and Kang et al. (2021).

Overall, Table 3 illustrates that the diversity of imaging modalities, equipment, and parameter settings demonstrates the adaptability of AI‐enabled bacterial imaging but also reveals the lack of standardized protocols for optimizing pathogen visualization.

##### 3.2.2.3 Data Preprocessing and AI Analysis Details

Preprocessing techniques varied widely to address tasks such as data quality control, region of interest (ROI) segmentation, and feature extraction, as summarized in Table 4. Baseline steps included resizing images to align with deep learning input requirements, followed by normalization or standardization to adjust feature scales, reduce bias, and ensure consistent contributions during model training. Spectral data preprocessing as part of hyperspectral microscopy data analysis frequently incorporated Savitzky–Golay smoothing for noise reduction, standard normal variate (SNV) for scatter correction, and multiplicative scatter correction or mean centering to minimize illumination variability (Fong et al. 2020; Kang et al. 2020a, 2020b, 2020c, 2021; Park et al. 2023; Seo et al. 2013; Signoroni et al. 2018; Turra et al. 2017; Wu et al. 2024). Some studies also reported background subtraction, flat‐field correction, or spectral band selection based on principal component loadings to improve signal‐to‐noise ratio and computational efficiency.

To expand biological datasets and focus on individual cells or cellular features, 5 studies divided images into smaller patches (Ali et al. 2020; Borowa et al. 2021, 2023; Hoorali et al. 2020; Spahn et al. 2022), while 16 studies applied ROI segmentation with various techniques (e.g., manual selection, thresholding, watershed, *k*‐means clustering, or U‐Net) (Fong et al. 2020; Gu et al. 2020; He et al. 2018; Kang et al. 2020a, 2020b, 2020c, 2021; Maeda et al. 2018; Park et al. 2023; Y. W. Seo et al. 2013; Signoroni et al. 2018; Spahn et al. 2022; Tao et al. 2023; Turra et al. 2017; Wu et al. 2024; Zhu et al. 2014). In addition, standard image augmentation was reported in 11 studies, including geometric transformations (e.g., rotation, flipping, scaling, shifting, cropping, shearing, zooming) and photometric adjustments (color, brightness, contrast, hue) to increase dataset size and improve model generalization (Akbar et al. 2021; Borowa et al. 2021, 2023; Hoorali et al. 2020; Ibrahim et al. 2021; Kang et al. 2020b; Ma et al. 2023; Rattray et al. 2023; Spahn et al. 2022; Wu et al. 2024; Yi et al. 2023).

The included studies employed a wide variety of AI model architectures, reflecting the breadth of approaches (Table 4). Principal component analysis (PCA) for dimensionality reduction was commonly coupled with traditional ML classifiers in 12 studies. In these studies, classifiers were trained on engineered feature sets derived from the images or multimodal images, including spectral reflectance values at selected wavelengths, PCA scores, intensity statistics, morphological descriptors (e.g., colony size, shape, or perimeter). Most often traditional ML classifiers included *k*‐nearest neighbors (*k*‐NN), support vector machine (SVM), or random forest (RF), either as primary models or as comparative benchmarks in 15 studies. Deep learning approaches, including convolutional neural networks (CNNs) and recurrent neural networks (RNNs), were employed in 23 studies. Common CNN architectures included U‐Net for ROI segmentation (e.g., single‐cell, colony), as well as historically state‐of‐the‐art models for image classification (e.g., AlexNet, VGG, ResNet, Xception, MobileNet, DenseNet, EfficientNet) for classifying whole images, patches, or ROIs. In addition, object detection models (e.g., YOLO and R‐CNN series) were applied, facilitating bacterial enumeration in addition to detection and identification (Ma et al. 2023; Yi et al. 2023). RNN architectures, particularly long short‐term memory (LSTM) networks, handled temporal or spectral sequence data (Fong et al. 2020; Kang et al. 2020; Kang et al. 2021; Wu et al. 2024), while multiple instance learning (MIL) frameworks were adapted for weakly labeled imaging datasets (Borowa et al. 2021, 2023).

Several studies employed various validation approaches to ensure model robustness and generalizability. The most common method was hold‐out validation, where datasets were initially divided into training and test sets using standard split (i.e., 70/30, 80/20, 85/15), and then further partitioned to include an additional hold‐out validation set to prevent overfitting during training (Hoorali et al. 2020; Kang et al. 2020a, 2020b, 2020c, 2021; Ma et al. 2023; Park et al. 2023; Rattray et al. 2023; Yi et al. 2023). Other studies used shuffle‐and‐split cross‐validation (i.e., repeated hold‐out validation) (Signoroni et al. 2018; Turra et al. 2017). For simpler statistical or traditional ML models, leave‐one‐replicate‐out cross‐validation (i.e., full cross‐validation) (Ali et al. 2020; Gu et al. 2020; Maeda et al. 2018) or *k*‐fold cross‐validation (He et al. 2018) was used. Although unusual due to its computational cost, some studies applied *k*‐fold cross‐validation to CNNs models (Borowa et al. 2021; Ibrahim et al. 2021). Notably, one study utilized a fully independent, unseen test set originating from distinct sources to assess model performance against more realistic samples (Yi et al. 2023). However, some studies either did not explicitly mention the use of an unseen test set beyond cross‐validation or lacked a separate validation step altogether (Akbar et al. 2021; Borowa et al. 2021; He et al. 2018; Maeda et al. 2018; Signoroni et al. 2018; Treebupachatsakul and Poomrittigul 2019, 2020; Turra et al. 2017; Wu et al. 2024).

Overall, the diversity of preprocessing pipelines, segmentation approaches, model architectures, and validation strategies demonstrates the adaptability of AI‐enabled bacterial imaging across diverse datasets and objectives, but also underscores the absence of standardized workflows for benchmarking performance and optimizing pathogen visualization.

#### 3.2.3 Comparator Characteristics

In this systematic review, comparators were defined as conventional detection methods that did not involve AI algorithms for pathogen classification, such as culture‐based plating or molecular assays. Table S3 provides details on the comparators used in each study. Out of the 28 included studies, only 3 reported using a comparator as a control to evaluate the AI‐enabled detection methods. Of these, two studies used traditional plating for bacterial detection and enumeration (Ma et al. 2023; Yi et al. 2023), with Yi et al. (2023) also including quantitative polymerase chain reaction (qPCR). In one study, a team of microbiologists analyzed the same dataset used for model training and test, serving as ground truth labels (Ibrahim et al. 2021). For the two studies that used plating or qPCR, the comparator was applied to the same sample preparation as the AI‐enabled imaging (“same as index”), ensuring direct comparability. Only Yi et al. (2023) reported a detection limit for its comparator.

Across the remaining 25 studies, comparator characteristics were largely NR for method type, sample preparation, target detection level, and detection limit, limiting the ability to assess performance differences. Notably, many expected reference methods, such as culture enrichment, biochemical assays, or immunoassays, were absent from the descriptions. The infrequent and inconsistent use of comparators underscores a critical gap in current AI‐enabled bacterial imaging research, as it prevents standardized benchmarking against established detection workflows.

#### 3.2.4 Pathogen Detection Performance

Across the included studies, there was substantial variability in reporting pathogen detection performance. Table 5 shows the most common evaluation metrics used in AI model evaluation, including classification accuracy, sensitivity, specificity, and area under the curve (AUC) values. In AI/ML benchmarking, a threshold of 70% is generally considered acceptable across these metrics. Values above 90% are typically regarded as high but optimal values vary by applications. However, extremely high accuracy or AUC close to 1 may indicate overfitting, particularly in studies using small or highly controlled datasets where the model may fail to generalize to real‐world data.

Classification accuracy is the ratio of correctly predicted instances (i.e., true positive and true negative) to the total number of instances (i.e., all possible classifications). It is calculated as follows:

(1) $$\mathrm{Accuracy}\;=\frac{\mathrm{TP}+\mathrm{TN}}{\mathrm{TP}\;+\;\mathrm{TN}\;+\;\mathrm{FP}\;+\;\mathrm{FN}}\;\times 100$$

where TP is true positive, TN is true negative, FP is false positive, and FN is false negative. Reporting an accuracy alone can be misleading, especially in imbalanced datasets where one class dominates. This metric ranged broadly from as low as 56.3% (Borowa et al. 2021) to as high as 100% (Maeda et al. 2018; Park et al. 2023; Y. W. Seo et al. 2013; Yi et al. 2023). Most studies consistently reported classification accuracy above 70%, demonstrating the potential of AI in imaging‐based pathogen detection despite differences in approach. Studies achieving 100% accuracy were often conducted in controlled laboratory environments, where overfitting is a likely concern. However, none of the included studies explicitly discussed the potential implications of overfitting in their analyses. Studies with lower accuracies tended to involve more complex, real‐world datasets or broader classification challenges.

Fewer studies reported sensitivity (i.e., TP rate or recall) and specificity (i.e., TN rate) metrics. Sensitivity measures how well the model identifies actual positives as follows:

(2) $$\mathrm{Sensitivity}\;=\frac{\mathrm{TP}}{\mathrm{TP}\;+\;\mathrm{FN}}\;\times 100$$

In the included studies, sensitivity values ranged from as low as 36% (Spahn et al. 2022) to 100% (Ma et al. 2023; Maeda et al. 2018; Spahn et al. 2022). Specificity, on the other hand, measures how well the model identifies actual negatives as follows:

(3) $$\mathrm{Specificity}\;=\frac{\mathrm{TN}}{\mathrm{TN}\;+\;\mathrm{FP}}\;\times 100$$

Specificity values in the included studies ranged from 80% (Hoorali et al. 2020) to 100% (Ibrahim et al. 2021; Maeda et al. 2018). These metrics highlight the variability in the performance of AI models depending on dataset composition and the complexity of pathogen detection tasks. The pathogen detection task type, which often involves distinguishing between infected and non‐infected samples, was commonly associated with these metrics.

Beyond absolute values, the selection of evaluation metrics often reflected the specific task and imaging approach. Binary classification tasks, such as detecting the presence or absence of a pathogen, most frequently reported accuracy, sensitivity, and specificity. Multi‐class classification tasks, including those distinguishing between multiple species or strains, sometimes incorporated macro‐averaged F1‐scores or kappa coefficients to account for imbalanced datasets. Object detection and segmentation studies (e.g., Ma et al. 2023; Spahn et al. 2022) were more likely to report intersection over union (IoU), mean average precision (mAP), or structural similarity (SSIM), which better capture spatial localization and object‐level detail.

AUC values, which reflect the balance between sensitivity and specificity, provide an overall picture of model performance. They are derived from the receiver operating characteristic (ROC) curve, which plots the TP rate (i.e., sensitivity) against the false positive rate (e.g., 1–specificity) across different classification thresholds. AUC values range from 0 and 1, with values closer to 1 indicating better discrimination between positive and negative cases. AUC values in the included studies ranged from 68.3% (Borowa et al. 2021) to 100% (Kang et al. 2020c; H. Zhu et al. 2023). Studies using controlled imaging conditions, often with high signal‐to‐noise and well‐separated classes, tended to report AUC and accuracy above 90%, while those using heterogeneous, real‐world samples showed greater variability, with performance occasionally dropping below 70%.

Notably, 9 of the 28 studies reported only a single metric. This lack of standardized, multi‐metric reporting makes it difficult to benchmark AI models across different optical platforms, sample types, and computational pipelines. Establishing a minimal reporting standard that includes accuracy, sensitivity, specificity, and AUC (or task‐specific equivalents) would improve reproducibility and facilitate cross‐study comparisons. In summary, while many studies reported high performance metrics, variability in evaluation methods and incomplete reporting limit the ability to assess generalizability and to draw robust comparisons across different AI‐enabled pathogen detection approaches.

### 3.3 Risk of Bias Assessment

**FIGURE 3:**
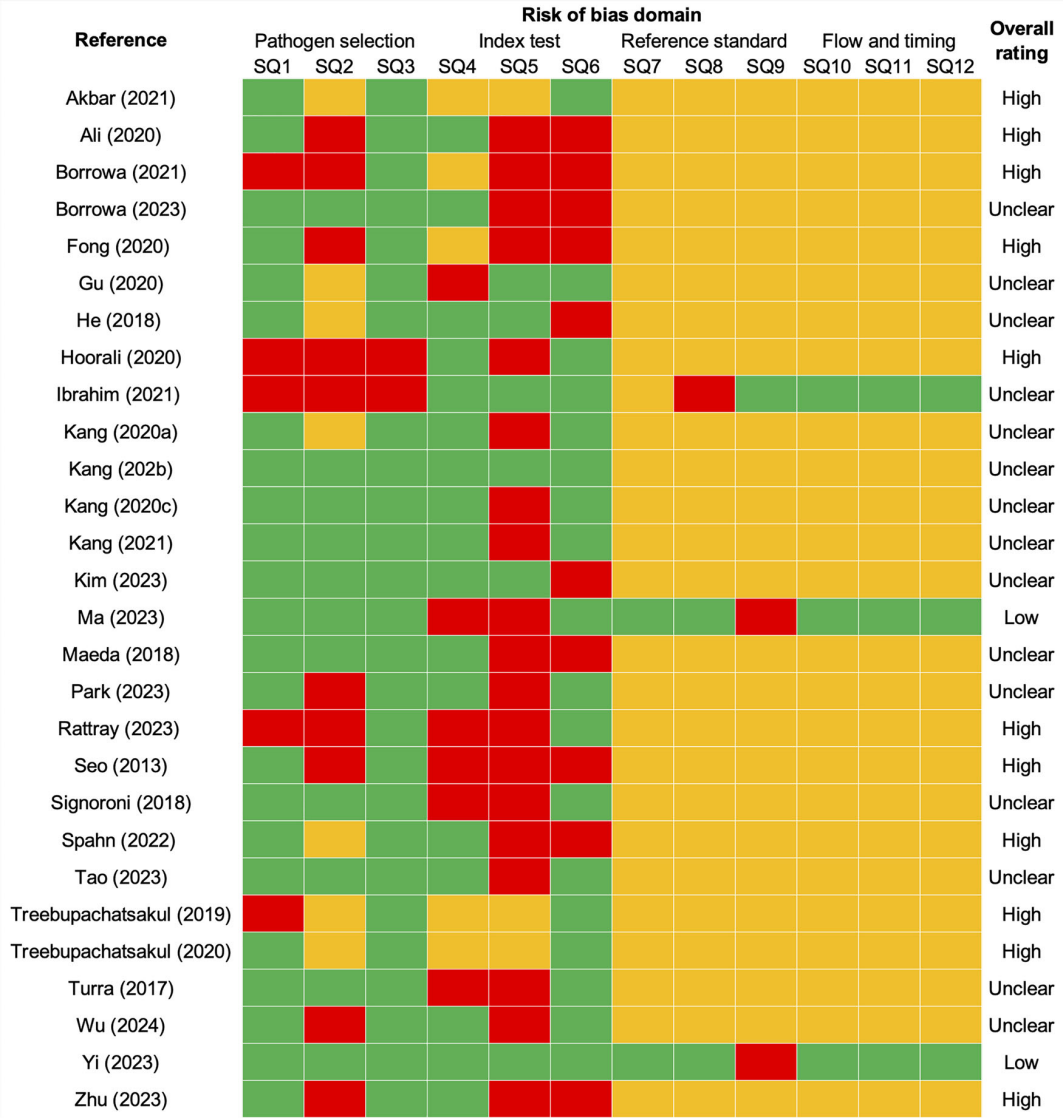
Summary of risk of bias assessments for the 28 included studies using the modified QUADAS‐2 criteria with 12 signaling questions (SQs) detailed in Table S2. Ratings: green (yes), yellow (unclear), and red (no). Studies were rated as “high” (< 4 yes), “low” (> 8 yes), or “unclear.” (4–8 yes) risk of bias.

Figure 3 summarizes the risk of bias assessment for the 28 included studies using the modified QUADAS‐2 tool, which was tailored to the specific context of AI‐enabled imaging‐based pathogen detection. As detailed in Section 2.6, the 12 signaling questions were grouped into four domains: pathogen selection, index test (i.e., proposed AI‐enabled detection methods), reference standard (i.e., conventional detection methods), and flow and timing. Each cell in the heatmap reflects whether a study met a given signaling question (“yes” = green, “no” = red, “unclear” = yellow). The overall risk of bias rating for each study was determined based on the distribution of responses across all domains. Out of the 28 included studies, 11 were rated as “high risk of bias”, often due to limited reporting on bacterial strain accessibility, lack of clarity in preprocessing workflows, or missing details on reference standard comparators. Fifteen studies were rated as “unclear” risk, typically because essential methodological details were not reported. Only two studies achieved a “low risk of bias” rating, reflecting comprehensive transparency across all assessed domains. These ratings reflect internal validity and reporting sufficiency as defined by QUADAS‐2 and should not be interpreted as a ranking of overall scientific importance or study quality.

Notably, the “index test” domain (SQ4–SQ6) showed the highest proportion of “no” responses, indicating frequent gaps in describing whether AI‐enabled detection results were interpreted without knowledge of the reference standard, whether data preprocessing was implemented dynamically within the code rather than hardcoded, and whether data splitting and final dataset sizes were clearly explained. The “reference standard” domain (SQ7–SQ9) often lacked detail on whether the conventional method was itself likely to correctly classify bacterial samples, whether its results were interpreted independently of the index test, and whether it used comparable sample preparation methods and targeted the same cellular level as the index test. In the “flow and timing” domain (SQ10–SQ12), issues were observed with ensuring bacterial samples were in a consistent state before both tests, verifying that all test set samples underwent the reference standard, and reporting whether the index test achieved comparable or faster turnaround times than the reference method. Collectively, these gaps underscore the need for more standardized and transparent reporting in AI‐enabled pathogen detection research, particularly in how data are prepared, interpreted, and timed, to support replication, meta‐analysis, and real‐world application.

## 4 Discussion

In this systematic review, we revisited four key RQs: (1) identifying AI algorithms employed in imaging for pathogen classification, (2) evaluating how AI enhances the speed and accuracy of pathogen detection compared to traditional methods, (3) highlighting the recent advancements in the integration of AI with multimodal imaging for pathogen research, and (4) exploring how AI techniques facilitate early detection of pathogens under stress conditions. Consistent with the small number of eligible studies identified in this systematic review, our findings indicate that AI‐enabled imaging for foodborne pathogen detection, particularly in food matrices, remains at an early stage. Although not all included studies were conducted directly with foodborne pathogens or in food matrices, they focused on bacterial pathogens and imaging conditions at the microbial level that are relevant to food safety applications. In many cases, model architectures and imaging pipelines were developed first in environmental or biomedical microbiology settings, but the underlying task of classifying bacterial cells or colonies is directly applicable to foodborne pathogens. By systematically mapping these approaches across neighboring domains, this review aims to provide food safety researchers with transferable AI‐enabled imaging strategies while also highlighting where dedicated validation in food matrices and real production environments is still needed.

Across the 28 included studies, the QUADAS‐2 assessment indicated that many investigations had high or unclear risk of bias (Figure 3), primarily due to incomplete reporting of sampling strategies, preprocessing steps, or reference methods. These reporting and methodological gaps limited our ability to directly compare diagnostic performance and precluded a formal meta‐analysis. To reduce such bias in future work, studies should clearly describe how samples are selected, how AI‐enabled and reference methods are implemented, and how timing and flow between tests are handled, so that diagnostic accuracy can be interpreted consistently and combined across studies when appropriate. More detailed recommendations on experimental and microbiological reporting are discussed in Section 4.1. In addition, because our search focused on peer‐reviewed journal articles in selected databases and did not systematically include gray literature (e.g., technical reports or theses), publication bias and incomplete coverage of relevant studies cannot be ruled out.

### 4.1 There Is No One‐Size‐Fits‐All Algorithm for AI‐Enabled Pathogen Classification

The included studies demonstrated that diverse AI models, ranging from traditional ML to advanced CNNs, have successfully achieved high pathogen classification performance by capturing spatial, spectral, and contextual features (Table 5). However, the effectiveness of these models is highly dataset‐tailored, with no single model architecture consistently superior across all scenarios. Data preprocessing techniques were frequently used to improve data quality and extract relevant features, and to align with input requirements of the state‐of‐the‐art model architectures (Table 4). Similar to the selection of AI model architectures, studies utilized diverse combinations of preprocessing techniques, further highlighting the context‐dependent nature of AI‐enabled pathogen detection. In particular, multimodal imaging produces high‐dimensional and heterogeneous datasets characterized by distinct modalities, scales, and feature representations, making thorough and tailored preprocessing critical (Cozzolino et al. 2023; Fong et al. 2020; Kang et al. 2020b; Park et al. 2023; Tao et al. 2023). Consequently, effective pathogen classification relies heavily on case‐specific optimization of preprocessing methods and AI model architectures. In contrast to much of the computer vision literature, where highly structured benchmark datasets (e.g., ImageNet or COCO) are used to evaluate model architectures under standardized and noise‐controlled conditions, AI studies in food safety must account for biological variability and heterogeneous sample conditions. This reflects the different end goals of the two fields: computer vision often focuses on comparing and optimizing computational efficiency, whereas food safety applications require models and training workflows that are robust to strain differences, growth conditions, and real‐world biological variability. As a result, experimental design, transparent reporting, and contextual metadata are especially important for AI model development and evaluation in this domain.

Building on these overall findings, this review also points to several specific reporting priorities at the study level. More standardized and transparently documented laboratory protocols, including bacterial incubation conditions and concentrations, imaging settings, and data size, would significantly improve reproducibility and enable meaningful comparisons across studies. For instance, basic microbiological factors such as whether cells are freshly cultured or recovered from frozen stocks, prior exposure to sanitizer or other stressors, and the choice of growth medium directly influence cell physiology and the resulting image signals, so they should be treated as core elements of data quality rather than minor technical details (Davey 2011; McGoverin, et al. 2021). Over time, developing AI‐enabled imaging workflows that are explicitly linked to established culture‐based methods could provide practical standards that support cross‐study comparison. Rather than prescribing a single incubation regime, we recommend that future work relate incubation choices to recognized microbiological guidelines where appropriate and report them in sufficient detail so that AI performance can be interpreted in the context of strain, incubation history, and imaging conditions.

Moreover, while early studies predominantly emphasized achieving high accuracy, greater attention should be devoted to the entire computational pipeline. Specifically, future research should carefully address overfitting and generalization by adopting validation methods, such as hold‐out validation with train/validation/test splits (Hoorali et al. 2020; Kang et al. 2020a, 2020b, 2020c, 2021; Ma et al. 2023; Park et al. 2023; Rattray et al. 2023), cross‐validation strategies (Ali et al. 2020; Gu et al. 2020; Ibrahim et al. 2021), or evaluation using fully independent test set from distinct sources (Yi et al. 2023). An unbiased assessment of model performance on unseen data can only be ensured by utilizing an independent test set that remains strictly separate from model training to prevent data leakage, thereby achieving genuine evaluation beyond the model‐fitting stage. Class imbalance is another important factor in data science, often overlooked in the included studies. This is particularly critical in object detection tasks, even when starting with a balanced number of images per class (Ma et al. 2023; Yi et al. 2023) due to severe foreground‐to‐background imbalance and sparse or small object instances within images. This inherent imbalance requires specialized approaches (e.g., focal loss, tailored anchors) to ensure accurate detection of underrepresented or less prominent objects.

Furthermore, establishing standardized benchmarks is essential for rigorous and consistent evaluation of AI‐enabled pathogen detection models. Computationally, this includes building open‐source AI communities around publicly accessible food safety and agricultural repositories hosting benchmark datasets (Joshi et al. 2023; Qian et al. 2025). Biologically, it involves adopting standardized reference protocols for pathogen detection, such as traditional plating methods and qPCR assays. Future research should also emphasize incorporating diverse real‐world samples to enhance the generalizability and applicability of AI‐enabled detection methods. As AI‐enabled systems continue to evolve, fostering interdisciplinary collaboration between microbiologists, imaging specialists, and data scientists, and sharing datasets, imaging protocols, and algorithms will be critical for translating experimental successes into robust, interoperable, and field‐ready frameworks.

### 4.2 Pathogen Detection Accelerated and Automated by AI Modeling

A common motivation across the included studies was the need for rapid pathogen detection methods, addressing limitations such as lengthy biological sample preparation and reliance on selective enrichment steps (Y. W. Seo et al. 2013; Maeda et al. 2018; Treebupachatsakul and Poomrittigul 2019; Ibrahim et al. 2021; Kang et al. 2021; Ma et al. 2023). Most included studies involving non‐AI comparator methods utilized culture‐based plating method, considered the gold standard (Table S3). In contrast, AI‐enabled imaging methods were investigated for their potential to substantially accelerate detection by reducing biological sample preparation time and automating complex data analysis workflows. Notably, several studies demonstrated that AI image classification achieved high accuracies using bacterial samples with less than 24 h of incubation, highlighting the strong potential of these methods for near real‐time pathogen monitoring (Ali et al. 2020; Borowa et al. 2023; Gu et al. 2020; He et al. 2018; Kim et al. 2023; Ma et al. 2023; Maeda et al. 2018; Park et al. 2023; Y. W. Seo et al. 2013; Signoroni et al. 2018; Spahn et al. 2022; Treebupachatsakul and Poomrittigul 2019; Turra et al. 2017; Wu et al. 2024; Yi et al. 2023). This represents a significant improvement compared to traditional techniques, which typically require several days for accurate detection (Foddai and Grant 2020).

A key strength of AI‐enabled pathogen detection approaches lies in their ability to streamline workflows and reduce dependence on pathogen‐specific enrichment steps. Studies that used generic media without selective enrichment made use of the AI model's capability to learn subtle differences in appearance among the bacteria tested, supporting broader and more adaptable detection capabilities. In addition, AI‐enabled imaging allows pathogen detection across multiple scales, from single‐cell to microcolony and colony levels, further reducing wait times compared to traditional methods. In particular, Ma et al. (2023) and Yi et al. (2023) demonstrated rapid pathogen detection capabilities, achieving classification within 4 h with accuracies above 90%. The speed and accuracy of AI models have broad application potentials in food safety, ranging from identifying contamination sources to predicting pathogen presence and cross‐contamination risks.

Despite these advantages, further testing and technical advancements are needed for the real‐world application of AI‐enabled pathogen detection. In many cases, AI models were trained and tested on relatively small datasets collected under controlled laboratory conditions. When exposed to real‐world variability, such as diverse food matrices and natural microbiota, model performance may decline. In addition, the black‐box nature of many AI models often lacks transparency in how predictions are made, raising concerns about the trustworthiness of results, especially in food safety regulatory contexts. Techniques such as explainable AI (XAI) can enhance model interpretability by providing contextual insight into model predictions (Hassija et al. 2024). While Table 4 shows that current work is dominated by CNNs, PCA‐based pipelines, and other supervised learning approaches, none of the studies included in this systematic review reported dedicated XAI techniques, and most relied primarily on global performance metrics to evaluate model quality. Although XAI methods have begun to appear in a small subset of the broader AI imaging literature, their adoption remains limited within pathogen detection applications (Ding et al. 2025). For food safety applications, XAI methods such as feature importance visualizations, class activation or saliency maps, and local surrogate models (e.g., local interpretable model‐agnostic explanations [LIME]) could help clarify which spatial or image‐derived features drive model decisions, thereby improving transparency for operators and regulators (Dwivedi et al. 2023). We identify the limited adoption of such techniques in the existing literature as an important opportunity for future research.

As these technical and interpretability challenges are addressed, integrating multimodal imaging and AI‐enabled detection systems into production environments could allow food safety programs to shift from periodic sampling, often reliant on third‐party laboratories, to more continuous in‐house monitoring with rapid detection capabilities. This shift has the potential to shorten response times and generate richer data streams for verification and monitoring. Such data could enhance traceability and contribute to a more comprehensive, automated audit trail consistent with Hazard Analysis and Critical Control Point (HACCP) principles and aligned with FDA expectations for documentation, transparency, and system validation.

### 4.3 Enhancing Capabilities of Multimodal Imaging Through AI Integration

AI‐enabled pathogen detection can benefit from multimodal imaging approaches that capture complementary structural and biochemical information, including detailed bacterial appearance, in a single dataset. Among these, hyperspectral imaging provides a non‐invasive approach that generates comprehensive pathogen signatures across spatial and spectral domains (Dale et al. 2013, Kang et al. 2020b). Unlike single‐modality imaging methods that primarily provide spatial information only, multimodal imaging offers richer, high‐dimensional data by combining spectral details with spatial context (Eady and Park 2016). This comprehensive approach allows for the detection of subtle pathogen characteristics, such as variations in intracellular structures and metabolic profiles, which may remain undetected with single‐modality techniques (Barzan et al. 2021; Piqueras et al. 2011). When integrated with advanced deep learning algorithms, multimodal datasets can further reveal subtle biological variations among bacterial species and strains (Tables 4 and 5). Park et al. (2023) demonstrated a major strength of this approach, using AI‐enabled hyperspectral microscopy to successfully differentiate between live and dead *Salmonella*, highlighting the potential for AI models to distinguish pathogens based on physiological states rather than static appearance alone. This capability to move beyond classification based only on images and to infer metabolic viability is especially crucial in food safety and public health contexts, where the presence of stressed cells, such as viable but non‐culturable (VBNC) cells, can challenge the effectiveness of traditional detection methods (Papa, Wasit, et al. 2025). Such advancements could also enable classification at more granular levels, including bacterial serovars (Papa, Bhattacharya, et al. 2025). Given the extensive diversity within pathogens like *Salmonella*, acknowledging unique phenotypic and genotypic variations among different strains and serovars is crucial (Khan et al. 2024; Sabbagh et al. 2010; Uzzau et al. 2000). Thus, hyperspectral imaging has been extensively utilized across pathogen detection studies at multiple scales (Table 3), ranging from microbial colonies (macroscopic) to single‐cell characteristics (microscopic). Consequently, multimodal imaging studies typically report higher classification accuracies than those relying solely on single modality imaging (Fong et al. 2020; Kang et al. 2020c; Park et al. 2023; Tao et al. 2023).

Furthermore, dimensionality reduction techniques play an important role in extracting meaningful biological variation from high‐dimensional imaging data. PCA, one of the most widely adopted approaches for feature extraction and dimensionality reduction (Table 4), provides an efficient way to retain the most informative variance components while simplifying data structure. By reducing dimensional complexity before classification, PCA can improve pathogen classification accuracy and computational efficiency (Ram et al. 2024; Y. Seo et al. 2016). However, PCA is limited by its sensitivity to multicollinearity and reliance on linear transformations based on Euclidean distances, which may not fully capture the nonlinear relationships within multimodal data. To overcome these constraints, recent deep learning approaches, including end‐to‐end convolutional models and attention‐based architectures, are increasingly being explored for their ability to model complex, nonlinear interactions and dynamically prioritize relevant features (Hong et al. 2020; Li et al. 2024).

### 4.4 Emerging Applications of AI‐Enabled Imaging for Detecting Stressed Pathogens

In real food system environments, from preharvest through processing, storage, and distribution, bacterial pathogens are rarely present under ideal growth conditions. They are often exposed to stressors such as cold, heat, acidification, desiccation, sanitizers, and osmotic shifts, which can drive cells into sub‐lethally injured or VBNC states (Foddai and Grant 2020). Stressed cells may exhibit reduced metabolic activity, altered membrane integrity, surface restructuring, and increased morphological heterogeneity, all of which can impair their recovery on selective media or detection by conventional biochemical or molecular assays (Oliver 2010; Ramamurthy et al. 2014). These stress‐induced phenotypic changes also alter their bio‐optical signatures, suggesting that AI‐enabled imaging could play a key role in detecting pathogens that evade traditional culture‐based methods (Papa, Wasit, et al. 2025). However, as shown in Table 2, only a limited number of the included studies explicitly evaluated pathogen detection under defined stress conditions. Notably, three included studies demonstrated that AI models can discriminate stressed or non‐viable states from actively growing populations using imaging data, extending beyond conventional “viable and culturable” endpoints of culture‐based methods (Ali et al. 2020; Park et al. 2023; Wu et al. 2024).

Beyond culture‐based methods, molecular approaches such as polymerase chain reaction (PCR) and sequencing also face challenges when pathogens enter stress‐induced states such as sub‐lethally injured, persister, or VBNC forms (Emerson et al. 2017; Zeng et al. 2016). Under these conditions, reduced DNA yield and amplification efficiency lower detection sensitivity, and neither PCR nor sequencing distinguishes viable from dead cells or captures physiological stress responses (Emerson et al. 2017; Zeng et al. 2016). As a result, many current methods primarily measure the presence of target DNA rather than the physiological state of the population. In contrast, hyperspectral and other multimodal microscopy techniques capture rich bio‐optical signatures that combine image‐based spatial information with spectral information and reflect microbial morphology, biochemical composition, viability, and stress responses (Kang et al. 2020b; Park et al. 2023; Zhang et al. 2023). When coupled with AI, these imaging data can reveal complex patterns associated with different species, serovars, and physiological states, including stressed and VBNC cells. For instance, we recently reported that AI integrated with multimodal imaging accurately differentiate *E. coli* in its viable and culturable state from its VBNC state induced by low‐level antimicrobial stressors (Papa, Wasit, et al. 2025). Together, these findings support the potential of AI‐enabled imaging to capture bio‐optical signatures associated with stress responses. This makes AI‐enabled imaging a promising front‐end screening tool that can rapidly flag stressed or emergent pathogen subpopulations for confirmatory molecular testing and genomic characterization.

Stress responses are also influenced by strain‐level and lineage‐level differences in genetic background, which shape regulatory networks, cell surface structures, and extracellular matrices (Lozada‐Chavez et al. 2006; Schellhorn 2020). These genetic signatures can manifest as distinct phenotypes in microscopic images, such as differences in cell clustering, microcolony formation, or biofilm‐like structures. Building on this, deeper exploration of isolate‐level variation and geospatially distinct pathogen populations, each adapted to local environmental stressors, will be critical for advancing AI‐enabled pathogen detection (Cheng et al. 2019; Marmion et al. 2022). Linking imaging‐derived features with genomic or transcriptomic data could enable high‐throughput phenotypic screening to prioritize isolates or conditions for more detailed molecular characterization. Integrating these sources of variations into AI model development and evaluation pipelines could yield more generalizable models that maintain detection performance across stress conditions, strain backgrounds, food matrices, and environmental contexts. Enhancing datasets with well‐characterized stressed populations, diverse isolates, and realistic food system scenarios is therefore a promising future direction with the potential to improve detection accuracy, strengthen model generalizability, and enable near real‐time monitoring of stressed pathogens in food safety applications.

## 5 Conclusion

This systematic review explored AI‐enabled imaging techniques for pathogen detection, highlighting advances in applying AI to microbial imaging alongside existing challenges related to biological methodology inconsistencies and reporting practices. AI‐enabled imaging has demonstrated significant potential in enhancing classification accuracy and reducing detection time across diverse conditions. However, critical gaps remain due to multidisciplinary complexities, underscoring the need for improved standardization to enhance reproducibility, facilitate meta‐analysis, and strengthen generalization. Establishing consistent laboratory protocols (e.g., bacterial incubation conditions, imaging settings) and refining computational pipeline (e.g., contextual data preprocessing, overfitting mitigation, class imbalance correction) are crucial for meaningful cross‐study comparisons. In addition, the integration of AI with multimodal imaging has further advanced pathogen detection by leveraging high‐dimensional data, yet challenges persist in optimizing feature extraction and fusion strategies. Incorporating diverse biological variations, including stress condition studies, will be essential for enhancing model robustness and real‐world applicability. Standardizing computational benchmarks and biological reference protocols, while integrating multimodal imaging and diverse biological contexts, will significantly improve generalization and applicability, supporting broader adoption of AI‐enabled pathogen detection in food safety, public health, and environmental monitoring.

## Author Contributions

**MeiLi Papa**: investigation, formal analysis, writing – original draft, writing – review and editing, visualization. **Gillian Kuehnle**: investigation, writing – review and editing. **Yoo Jung (Erika) Oh**: methodology, writing – review and editing. **Jiyoon Yi**: conceptualization, methodology, writing – original draft, writing – review and editing, visualization, project administration, funding acquisition, supervision, validation.

## Conflicts of Interest

The authors declare no conflicts of interest.

## Supporting information




**Supplementary Materials**: crf370468‐sup‐0001‐tablesS1‐S3.docx
