# Supplementary material for: AI‐Enabled Imaging for Pathogen Detection Under Stress Conditions: A Systematic Review
Source: Compr Rev Food Sci Food Saf. 2026 Apr 22;25(3):e70468. doi: 10.1111/1541-4337.70468 (PMC13103521; doi:10.1111/1541-4337.70468)
Supplement: Supplementary file 1 — Supplementary Materials: crf370468‐sup‐0001‐tablesS1‐S3.docx [file CRF3-25-e70468-s001.docx]

**Supplementary Information for**

**AI-enabled imaging for pathogen detection under stress conditions: A systematic review**

**Names and e-mail addresses for all authors**

MeiLi Papa^1^ [papameil@msu.edu](mailto:papameil@msu.edu)

Gillian Kuehnle^1^  [kuehnleg@msu.edu](mailto:kuehnleg@msu.edu)

Yoo Jung (Erika) Oh^2^ [ohyoojun@msu.edu](mailto:ohyoojun@msu.edu)

Jiyoon Yi ^1^ [yijiyoon@mdu.edu](mailto:yijiyoon@mdu.edu)

**Author affiliation(s)**

^1^Department of Biosystems and Agricultural Engineering, Michigan State University, East Lansing, MI 48824, USA

^2^Department of Communication, Michigan State University, East Lansing, MI 48824, USA

**Contact information for Corresponding author**

Jiyoon Yi, 524 S. Shaw Ln, East Lansing, MI 48824, USA. Email: [yijiyoon@msu.edu](mailto:yijiyoon@msu.edu)

**Supplementary Table S1.** Inclusion and exclusion criteria guided by the populations, interventions, comparators, outcomes, study design (PICOS) framework (Schardt et al. 2007).

|  |  |  |  | Inclusion criteria | Exclusion criteria |
| --- | --- | --- | --- | --- | --- |
| P |  |  | Population | Studies focusing on bacteria or their physiological signals under stress conditions | Studies focusing on non-bacterial substrates |
| I |  |  | Intervention | Studies applying AI/ML techniques for bacterial detection using optical microscopy focusing on morphology based on original datasets | Studies using electron microscopy or non-imaging methods, not focusing on morphology, or using downloaded datasets |
| C |  |  | Comparison | Comparison of AI-enabled imaging with traditional methods | Studies focusing only on traditional detection methods without AI/ML integration |
| O |  |  | Outcome | Accuracy, speed, and/or predictive capabilities of AI/ML in bacterial detection | N/A |
| S |  |  | Study design | Original research articles from peer-reviewed journals or conference proceedings | Dissertations/theses, reviews, books/book chapters, conference abstracts without full text, editorials, news articles, posters, datasets, patents, proposals, or retracted publications |
|  |  |  | Timeframe | 2012–2024 | Outside of 2012–2024 |
|  |  |  | Language | English | Non-English |

AI: Artificial intelligence. ML: Machine learning. N/A: Not applicable.

**Supplementary Table S2.** Risk of bias assessment domains and signaling questions used in this systematic review based on a modified Quality Assessment of Diagnostic Accuracy Studies 2 (QUADAS-2) tool (Whiting et al. 2011)

| Domain | Signaling questions |
| --- | --- |
| Pathogen selection  Index test  Reference standard  Flow and timing | 1. Were the bacterial strains diverse? 2. Were the bacterial strains commonly available and easily replicable? 3. Were the bacteria from a controlled source? 4. Were the index test (i.e., AI-enabled detection) results interpreted without the knowledge of the reference stanard? 5. Was data preprocessing implemented dynamically wihin the code, rather than being hardcoded? 6. Was the data split and final data size clearly explained? 7. Is the reference standard (i.e., conventional detection) likely to correctly classify bacterial samples? 8. Were the reference standard results interpreted without the knowledge of the results of the index test? 9. Did the reference standard  use similar sample preparation methods and target the same cellular level as the index test? 10. Were the bacterial samples in a consistent status before the index test and the reference standard test? 11. Were all bacterial samples for test sets subjected to the reference standard? 12. Was the overall testing time for the indext test faster or similar to that of the reference standard? |

**Supplementary Table S3.** Summary of comparator characteristics

| **No.** | **Reference** | **Conventional methods** | **Sample preparation** | **Target detection level** | **Detection limit** |
| --- | --- | --- | --- | --- | --- |
| 1 | Akbar (2021) | NR | NR | NR | NR |
| 2 | Ali (2020) | NR | NR | NR | NR |
| 3 | Borowa (2021) | NR | NR | NR | NR |
| 4 | Borowa (2023) | NR | NR | NR | NR |
| 5 | Fong (2020) | NR | NR | NR | NR |
| 6 | Gu (2020) | NR | NR | NR | NR |
| 7 | He (2018) | NR | NR | NR | NR |
| 8 | Hoorali (2020) | NR | NR | NR | NR |
| 9 | Ibrahim (2021) | Expert labeling | Same as index | N/A | N/A |
| 10 | Kang (2020a) | NR | NR | NR | NR |
| 11 | Kang (2020b) | NR | NR | NR | NR |
| 12 | Kang (2020c) | NR | NR | NR | NR |
| 13 | Kang (2021) | NR | NR | NR | NR |
| 14 | Kim (2023) | NR | NR | NR | NR |
| 15 | Ma (2023) | Plating | Same as index | Colony | NR |
| 16 | Maeda (2018) | NR | NR | NR | NR |
| 17 | Park (2023) | NR | NR | NR | NR |
| 18 | Rattray (2023) | NR | NR | NR | NR |
| 19 | Seo (2013) | NR | NR | NR | NR |
| 20 | Signoroni (2018) | NR | NR | NR | NR |
| 21 | Spahn (2022) | NR | NR | NR | NR |
| 22 | Tao (2023) | NR | NR | NR | NR |
| 23 | Treebupachatsakul (2019) | NR | NR | NR | NR |
| 24 | Treebupachatsakul (2020) | NR | NR | NR | NR |
| 25 | Turra (2017) | NR | NR | NR | NR |
| 26 | Wu (2024) | NR | NR | NR | NR |
| 27 | Yi (2023) | Plating, qPCR | Same as index | Colony | 10^3^ CFU/mL |
| 28 | Zhu (2023) | NR | NR | NR | NR |

N/A: Not applicable. NR: Not reported. qPCR: Quantitative polymerase chain reaction.

**References**

Akbar, Son Ali, Kamarul Hawari Ghazali, Habsah Hasan, Zeehaida Mohamed, and Wahyu Sapto Aji. "An enhanced classification of bacteria pathogen on microscopy images using deep learning." In *2021 4th International Seminar on Research of Information Technology and Intelligent Systems (ISRITI)*, pp. 119-123. IEEE, 2021. <https://doi.org/10.1109/ISRITI54043.2021.9702809>

Ali, Nairveen, Johanna Kirchhoff, Patrick Igoche Onoja, Astrid Tannert, Ute Neugebauer, Jürgen Popp, and Thomas Bocklitz. "Predictive modeling of antibiotic susceptibility in *E. coli* strains using the U-Net network and one-class classification." *IEEE Access* 8 (2020): 167711-167720. <https://doi.org/10.1109/ACCESS.2020.3022829>

Borowa, Adriana, Dawid Rymarczyk, Dorota Ochońska, Monika Brzychczy-Włoch, and Bartosz Zieliński. "Deep learning classification of bacteria clones explained by persistence homology." In *2021 International Joint Conference on Neural Networks (IJCNN)*, pp. 1-8. IEEE, 2021. <https://doi.org/10.1109/IJCNN52387.2021.9533954>

Borowa, Adriana, Dawid Rymarczyk, Dorota Ochońska, Agnieszka Sroka-Oleksiak, Monika Brzychczy-Włoch, and Bartosz Zieliński. "Identifying bacteria species on microscopic polyculture images using deep learning." *IEEE Journal of Biomedical and Health Informatics* 27, no. 1 (2022): 121-130. <https://doi.org/10.1109/JBHI.2022.3209551>

Fong, Alexandre, George Shu, Barry McDonogh, and Bosoon Park. "Detecting foodborne pathogens with darkfield hyperspectral microscopy." In *Hyperspectral Imaging and Applications*, vol. 11576, pp. 9-16. SPIE, 2020. <https://doi.org/10.1117/12.2584913>

Gu, Peng, Yao-Ze Feng, Le Zhu, Li-Qin Kong, Xiu-ling Zhang, Sheng Zhang, Shao-Wen Li, and Gui-Feng Jia. "Unified classification of bacterial colonies on different agar media based on hyperspectral imaging and machine learning." *Molecules* 25, no. 8 (2020): 1797. <https://doi.org/10.3390/molecules25081797>

He, Yingchuan, Weize Xu, Yao Zhi, Rohit Tyagi, Zhe Hu, and Gang Cao. "Rapid bacteria identification using structured illumination microscopy and machine learning." *Journal of Innovative Optical Health Sciences* 11, no. 1 (2018): 1850007. <https://doi.org/10.1142/S1793545818500074>

Hoorali, Fatemeh, Hossein Khosravi, and Bagher Moradi. "Automatic *Bacillus* *anthracis* bacteria detection and segmentation in microscopic images using UNet++." *Journal of Microbiological Methods* 177 (2020): 106056. <https://doi.org/10.1016/j.mimet.2020.106056>

Ibrahim, Abdullahi Umar, Emrah Guler, Meryem Guvenir, Kaya Suer, Sertan Serte, and Mehmet Ozsoz. "Automated detection of *Mycobacterium* *tuberculosis* using transfer learning." *The Journal of Infection in Developing Countries* 15, no. 5 (2021): 678-686. <https://doi.org/10.3855/jidc.13532>

Kang, Rui, Bosoon Park, and Kunjie Chen. "Identifying non-O157 Shiga toxin-producing *Escherichia coli* (STEC) using deep learning methods with hyperspectral microscope images." *Spectrochimica Acta Part A: Molecular and Biomolecular Spectroscopy* 224 (2020a): 117386. <https://doi.org/10.1016/j.saa.2019.117386>

Kang, Rui, Bosoon Park, Matthew Eady, Qin Ouyang, and Kunjie Chen. "Classification of foodborne bacteria using hyperspectral microscope imaging technology coupled with convolutional neural networks." *Applied Microbiology and Biotechnology* 104, no. 7 (2020b): 3157-3166. <https://doi.org/10.1007/s00253-020-10387-4>

Kang, Rui, Bosoon Park, Matthew Eady, Qin Ouyang, and Kunjie Chen. "Single-cell classification of foodborne pathogens using hyperspectral microscope imaging coupled with deep learning frameworks." *Sensors and Actuators B: Chemical* 309 (2020c): 127789. <https://doi.org/10.1016/j.snb.2020.127789>

Kang, Rui, Bosoon Park, Qin Ouyang, and Ni Ren. "Rapid identification of foodborne bacteria with hyperspectral microscopic imaging and artificial intelligence classification algorithms." *Food Control* 130 (2021): 108379. <https://doi.org/10.1016/j.foodcont.2021.108379>

Kim, Min Jeong, Jinyong Park, Minjae Kang, Uidon Jeong, Dokyung Jeong, Nae-Gyu Kang, Seung Jin Hwang et al. "Bacteria detection and species identification at the single-cell level using super-resolution fluorescence imaging and AI analysis." *Biosensors and Bioelectronics* 240 (2023): 115603. <https://doi.org/10.1016/j.bios.2023.115603>

Ma, Luyao, Jiyoon Yi, Nicharee Wisuthiphaet, Mason Earles, and Nitin Nitin. "Accelerating the detection of bacteria in food using artificial intelligence and optical imaging." *Applied and Environmental Microbiology* 89, no. 1 (2023): e01828-22. <https://doi.org/10.1128/aem.01828-22>

Maeda, Yoshiaki, Yui Sugiyama, Atsushi Kogiso, Tae-Kyu Lim, Manabu Harada, Tomoko Yoshino, Tadashi Matsunaga, and Tsuyoshi Tanaka. "Colony fingerprint-based discrimination of *Staphylococcus* species with machine learning approaches." *Sensors* 18, no. 9 (2018): 2789. <https://doi.org/10.3390/s18092789>

Park, Bosoon, Taesung Shin, Bin Wang, Barry McDonogh, and Alexandre Fong. "Classification between live and dead foodborne bacteria with hyperspectral microscope imagery and machine learning." *Journal of Microbiological Methods* 209 (2023): 106739. <https://doi.org/10.1016/j.mimet.2023.106739>

Rattray, Jennifer B., Ryan J. Lowhorn, Ryan Walden, Pedro Márquez-Zacarías, Evgeniya Molotkova, Gabriel Perron, Claudia Solis-Lemus, Daniel Pimentel Alarcon, and Sam P. Brown. "Machine learning identification of *Pseudomonas* *aeruginosa* strains from colony image data." *PLoS Computational Biology* 19, no. 12 (2023): e1011699. <https://doi.org/10.1371/journal.pcbi.1011699>

Schardt, Connie, Martha B. Adams, Thomas Owens, Sheri Keitz, and Paul Fontelo. "Utilization of the PICO framework to improve searching PubMed for clinical questions." *BMC Medical Informatics and Decision Making* 7, no. 1 (2007): 16. <https://doi.org/10.1186/1472-6947-7-16>

Seo, Young Wook, Seung Chul Yoon, Bosoon Park, Arthur Hinton Jr, William R. Windham, and Kurt C. Lawrence. "Development of classification models to detect *Salmonella* Enteritidis and *Salmonella* Typhimurium found in poultry carcass rinses by visible-near infrared hyperspectral imaging." In *Sensing for Agriculture and Food Quality and Safety V*, vol. 8721, pp. 70-78. SPIE, 2013. <https://doi.org/10.1117/12.2016336>

Signoroni, Alberto, Mattia Savardi, Mario Pezzoni, Fabrizio Guerrini, Simone Arrigoni, and Giovanni Turra. "Combining the use of CNN classification and strength‐driven compression for the robust identification of bacterial species on hyperspectral culture plate images." *IET Computer Vision* 12, no. 7 (2018): 941-949. <https://doi.org/10.1049/iet-cvi.2018.5237>

Spahn, Christoph, Estibaliz Gómez-de-Mariscal, Romain F. Laine, Pedro M. Pereira, Lucas von Chamier, Mia Conduit, Mariana G. Pinho et al. "DeepBacs for multi-task bacterial image analysis using open-source deep learning approaches." *Communications Biology* 5, no. 1 (2022): 688. <https://doi.org/10.1038/s42003-022-03634-z>

Tao, Chenglong, Jian Du, Junjie Wang, Bingliang Hu, and Zhoufeng Zhang. "Rapid identification of infectious pathogens at the single-cell level via combining hyperspectral microscopic images and deep learning." *Cells* 12, no. 3 (2023): 379. <https://doi.org/10.3390/cells12030379>

Treebupachatsakul, Treesukon, and Suvit Poomrittigul. "Bacteria classification using image processing and deep learning." In *2019 34th International Technical Conference on Circuits/Systems, Computers and Communications (ITC-CSCC)*, pp. 1-3. IEEE, 2019. <https://doi.org/10.1109/ITC-CSCC.2019.8793320>

Treebupachatsakul, Treesukon, and Suvit Poomrittigul. "Microorganism image recognition based on deep learning application." In *2020 International Conference on Electronics, Information, and Communication (ICEIC)*, pp. 1-5. IEEE, 2020. <https://doi.org/10.1109/ICEIC49074.2020.9051009>

Turra, Giovanni, Simone Arrigoni, and Alberto Signoroni. "CNN-based identification of hyperspectral bacterial signatures for digital microbiology." In *International Conference on Image Analysis and Processing*, pp. 500-510. Cham: Springer International Publishing, 2017. <https://doi.org/10.1007/978-3-319-68548-9_46>

Whiting, Penny F., Anne WS Rutjes, Marie E. Westwood, Susan Mallett, Jonathan J. Deeks, Johannes B. Reitsma, Mariska MG Leeflang, Jonathan AC Sterne, Patrick MM Bossuyt, and QUADAS-2 Group. "QUADAS-2: a revised tool for the quality assessment of diagnostic accuracy studies." *Annals of Internal Medicine* 155, no. 8 (2011): 529-536. <https://doi.org/10.7326/0003-4819-155-8-201110180-00009>

Wu, Chenlu, Yanqing Xie, Qiang Xi, Xiangli Han, Zheng Li, Gang Li, Jing Zhao, and Ming Liu. "Rapid and high accurate identification of *Escherichia coli* active and inactivated state by hyperspectral microscope imaging combing with machine learning algorithm." *Vibrational Spectroscopy* 130 (2024): 103645. <https://doi.org/10.1016/j.vibspec.2023.103645>

Yi, Jiyoon, Nicharee Wisuthiphaet, Pranav Raja, Nitin Nitin, and J. Mason Earles. "AI-enabled biosensing for rapid pathogen detection: from liquid food to agricultural water." *Water Research* 242 (2023): 120258. <https://doi.org/10.1016/j.watres.2023.120258>

Zhu, He, Jing Luo, Jiaqi Liao, and Sailing He. "High-accuracy rapid identification and classification of mixed bacteria using hyperspectral transmission microscopic imaging and machine learning." *Progress In Electromagnetics Research* 178 (2023). <https://doi.org/10.2528/PIER23082303>
